# Supplementary material for: Identification and Isolation of α-Glucosidase Inhibitors from Siraitia grosvenorii Roots Using Bio-Affinity Ultrafiltration and Comprehensive Chromatography
Source: Int J Mol Sci. 2023 Jun 15;24(12):10178. doi: 10.3390/ijms241210178 (PMC10299727; doi:10.3390/ijms241210178)
Supplement: Supplementary file 1 [file ijms-24-10178-s001.zip › ijms-2411049-supplementary.pdf]

Identification and isolation of *α*-glucosidase inhibitors from *Siraitia grosvenorii* roots using bio-affinity ultrafiltration and comprehensive chromatography

Fenglai Lu 1,# , Jiayi Sun 1,#, Xiaohua Jiang 1, Jingru Song 1, Xiaojie Yan 1, Qinghu Teng 2,* and DianPeng Li 1,*

1 Guangxi Key Laboratory of Plant Functional Phytochemicals and Sustainable Utilization, Guangxi Institute of Botany, Guangxi Zhuang Autonomous Region and Chinese Academy of Sciences, Guilin 541006, China

2 Guangxi Key Laboratory of Electrochemical and Magnetochemical Functional Materials, College of Chemistry and Bioengineering, Guilin University of Technology, Guilin 541004, China

* Correspondence: phytoldp@hotmail.com (D. Li), 2020130@glut.edu.cn (Q. Teng)

# These authors contribute equally to this work and joint first authors.

Supplementary data

Extraction and Isolation

The SGR2 extract (38 g) was separated into five fractions (Fr 2.1- Fr2.5) on MCI gel column (60-230 mesh, 15 × 25 cm) and eluted with a solvent system of MeOH/H_2O (10% to 100%). Fr 2.1 was dissolved in the least amount of MeOH, diluted with 100 mL distilled water in a separating funnel and then extracted with ethyl acetate and then concentrated under reduced pressure to obtain the ethyl acetate partition (Fr 2.1 Y) and H_2O partition (Fr 2.1 S). Fraction Fr 2.1 Y was separated by high-speed countercurrent using chloroform/methanol/water (10:5.5:4.5, v/v) yielded compound **3** (16.1 mg, 0.000161%), and the mixture of compound 1 and 2. Then, hexane/ethyl acetate/methanol/water (3:7:3:7, v/v) was used as a solvent system for HSCCC

separation of the mixture fraction and produced compound **1**(25.8 mg, 0.000258%) and **2** (42.7 mg, 0.000427%). Fr 2.2 was fractionated using preparative HPLC (pre-HPLC) and eluted with 20% acetonitrile (ACN) in H_2O (flow rate: 3.0 mL/min) to produce compound **4** (18.6 mg, 0.000162%), **5** (30.7 mg, 0.000307%), **6** (15.0 mg, 0.00015%), and **7** (14.6 mg, 0.000146%). Fr 2.3 was separated using pre-HPLC and eluted with 20%-35% ACN (flow rate: 3.0 mL/min) to yield compound **8** (138.9 mg, 0.00139%). Fr 2.4 was purified using pre-HPLC and eluted with 20%-35% ACN (flow rate: 3.0 mL/min) to obtain compound **9** (14.3 mg, 0.000143%), **11** (36.7 mg, 0.000367%) and **12** (185.5 mg, 0.00186%). Fr 2.5 was extracted by hexane/ethyl acetate/methanol/water (3 : 7 : 3 : 7, v/v) obtained Fr 2.5 Y and Fr 2.5 S. Fraction Fr 2.5 Y was separated using HSCCC with n-hexane/ethyl acetate/methanol/water (4:6:5:5, v/v) to produce compound **13** (8.8 mg, 0.000088%), **15** (10.7 mg, 0.000107%), **16** (5.0 mg, 0.00005%), **17** (3.8 mg, 0.000038%). Fr 2.5 S was separated using pre-HPLC and eluted with 35% ACN (flow rate:3.0 mL/min) to produce compound **14** (10.8 mg, 0.000108%)

CONTENT

Figure S1. ^{1}H NMR spectrum of Siraitic acid III E (**4**) (500 MHz in pyridine-d5).
Figure S2. ^{13}C NMR spectrum of Siraitic acid III E (**4**) (125 MHz in pyridine-d5).
Figure S3. The HSQC spectrum of Siraitic acid III E (**4**).
Figure S4. The HMBC spectrum of Siraitic acid III E (**4**).
Figure S5. ^{1}H-^{1}H COSY spectrum of Siraitic acid III E (**4**).
Figure S6. The HREIMS spectroscopic data of Siraitic acid III E (**4**).
Figure S7. ^{1}H NMR spectrum of Siraitic acid IIb E (**6**) (500 MHz in pyridine-d5).
Figure S8. ^{13}C NMR spectrum of Siraitic acid IIb E (**6**) (125 MHz in pyridine-d5).
Figure S9. The HSQC spectrum of Siraitic acid IIb E (**6**).
Figure S10. The HMBC spectrum of Siraitic acid IIb E (**6**).
Figure S11. ^{1}H-^{1}H COSY spectrum of Siraitic acid IIb E (**6**).
Figure S12. The HREIMS spectroscopic data of Siraitic acid IIb E (**6**).
Figure S13. ^{1}H NMR spectrum of Siraitic acid II E (**7**) (500 MHz in pyridine-d5).
Figure S14. ^{13}C NMR spectrum of Siraitic acid II E (**7**) (125 MHz in pyridine-d5).
Figure S15 The HSQC spectrum of Siraitic acid II E (**7**).
Figure S16. The HMBC spectrum of Siraitic acid II E (**7**).
Figure S17. ^{1}H-^{1}H COSY spectrum of Siraitic acid II E (**7**).
Figure S18. The HREIMS spectroscopic data of Siraitic acid II E (**7**).
Figure S19. ^{1}H NMR spectrum of Siraitic acid IV H (**8**) (500 MHz in pyridine-d5).
Figure S20. ^{13}C NMR spectrum of Siraitic acid IV H (**8**) (125 MHz in pyridine-d5).

Figure S21 The HSQC spectrum of Siraitic acid IV H (**8**).
Figure S22. The HMBC spectrum of Siraitic acid IV H (**8**).
Figure S23. ^{1}H-^{1}H COSY spectrum of Siraitic acid IV H (**8**).
Figure S24. The HREIMS spectroscopic data of Siraitic acid IV H (**8**).
Figure S25. ^{1}H NMR spectrum of Siraitic acid II G (**9**) (500 MHz in pyridine-d5).
Figure S26. ^{13}C NMR spectrum of Siraitic acid II G (**9**) (125 MHz in pyridine-d5).
Figure S27 The HSQC spectrum of Siraitic acid II G (**9**).
Figure S28. The HMBC spectrum of Siraitic acid II G (**9**).
Figure S29. ^{1}H-^{1}H COSY spectrum of Siraitic acid II G (**9**).
Figure S30. The HREIMS spectroscopic data of Siraitic acid II G (**9**).
Figure S31. ^{1}H NMR spectrum of Siraitic acid II A (**11**) (500 MHz in pyridine-d5).
Figure S32. ^{13}C NMR spectrum of Siraitic acid II A (**11**) (125 MHz in pyridine-d5).
Figure S33 The HSQC spectrum of Siraitic acid II A (**11**).
Figure S34. The HMBC spectrum of Siraitic acid II A (**11**).
Figure S35. ^{1}H-^{1}H COSY spectrum of Siraitic acid II A (**11**).
Figure S36. The HREIMS spectroscopic data of Siraitic acid II A (**11**).

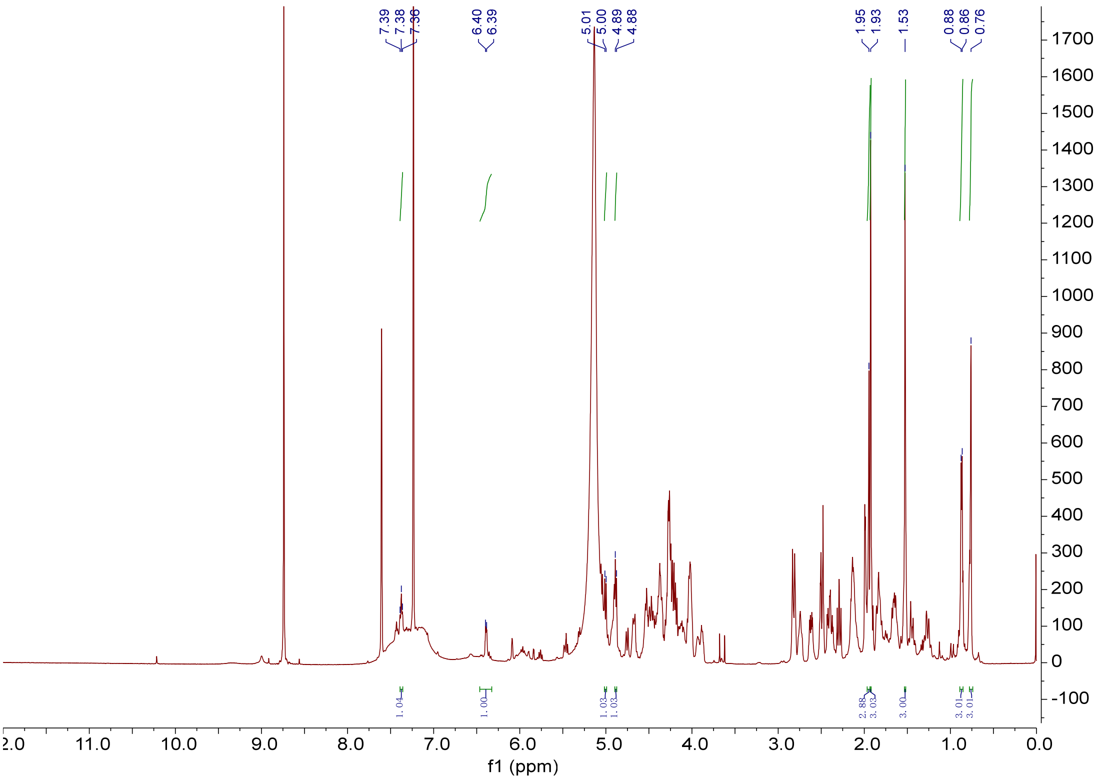

Figure S1. ^{1}H NMR spectrum of Siraitic acid III E (**4**) (500 MHz in pyridine-d5).

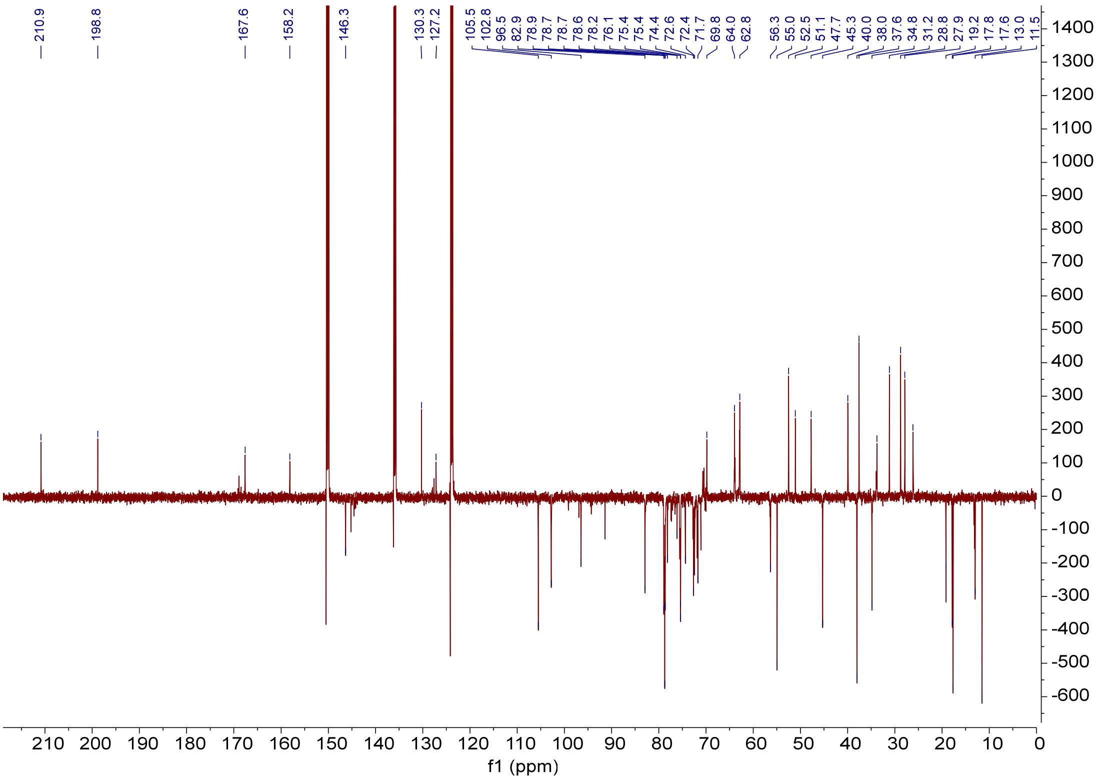

Figure S2. ^{13}C NMR spectrum of Siraitic acid III E (**4**) (125 MHz in pyridine-d5).

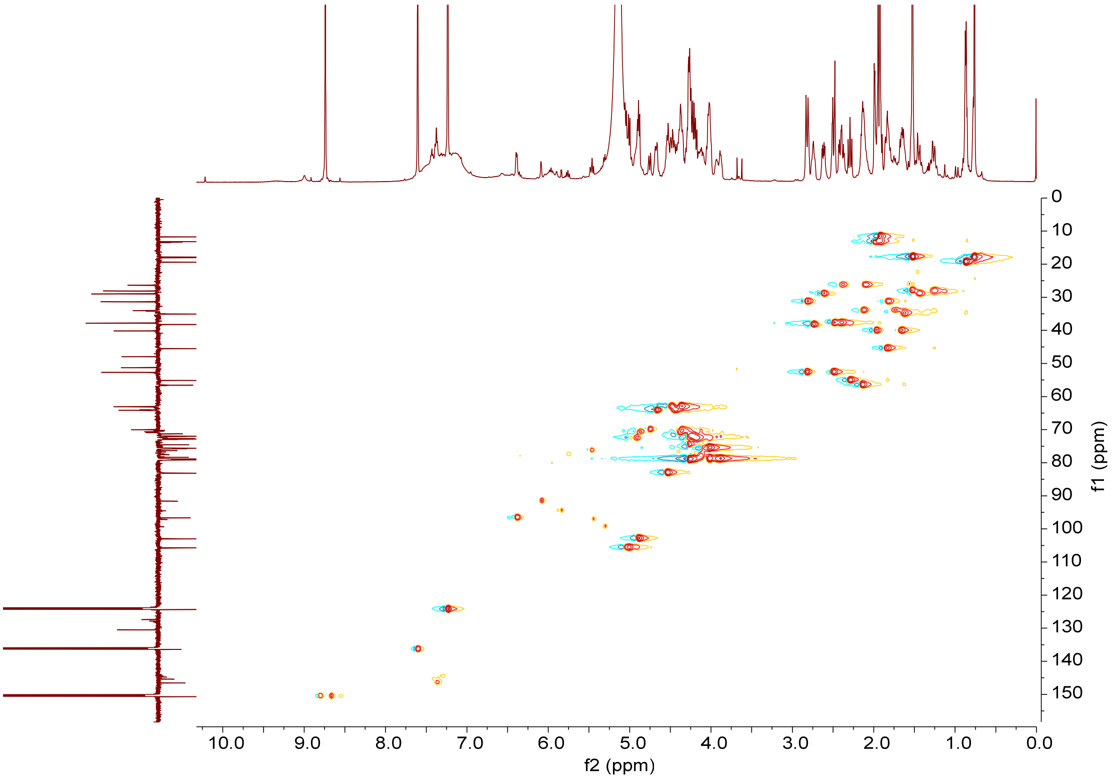

Figure S3. The HSQC spectrum of Siraitic acid III E (**4**).

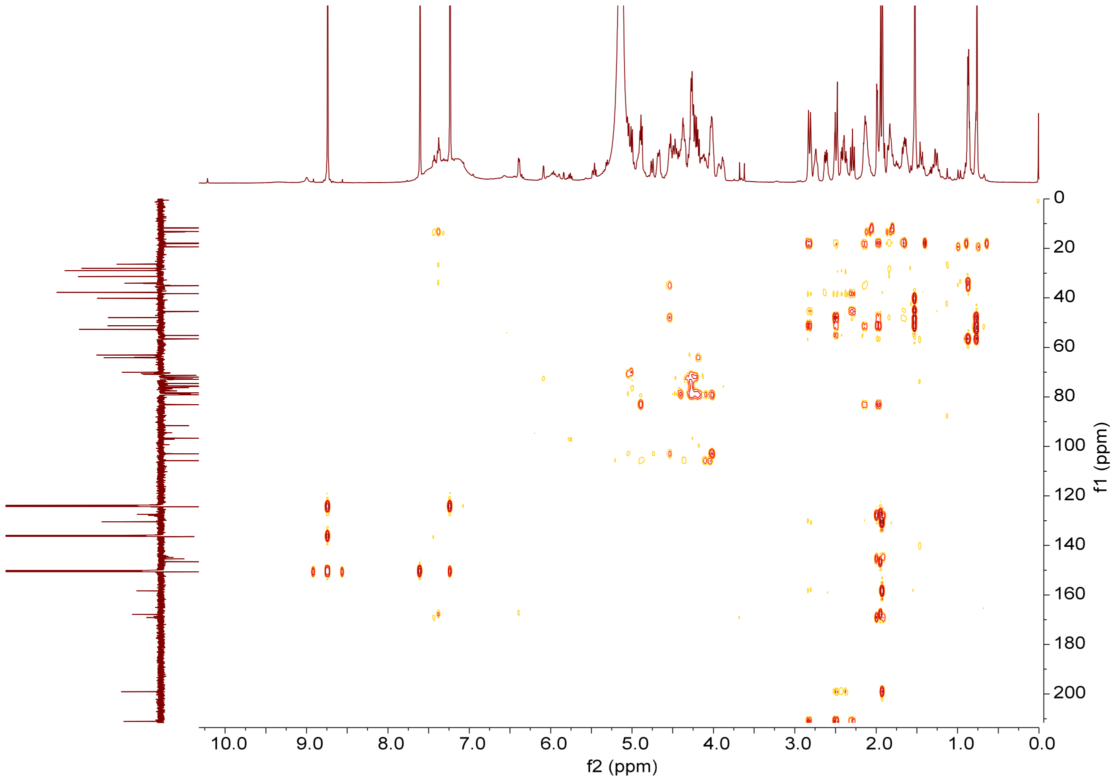

Figure S4. The HMBC spectrum of Siraitic acid III E (**4**).

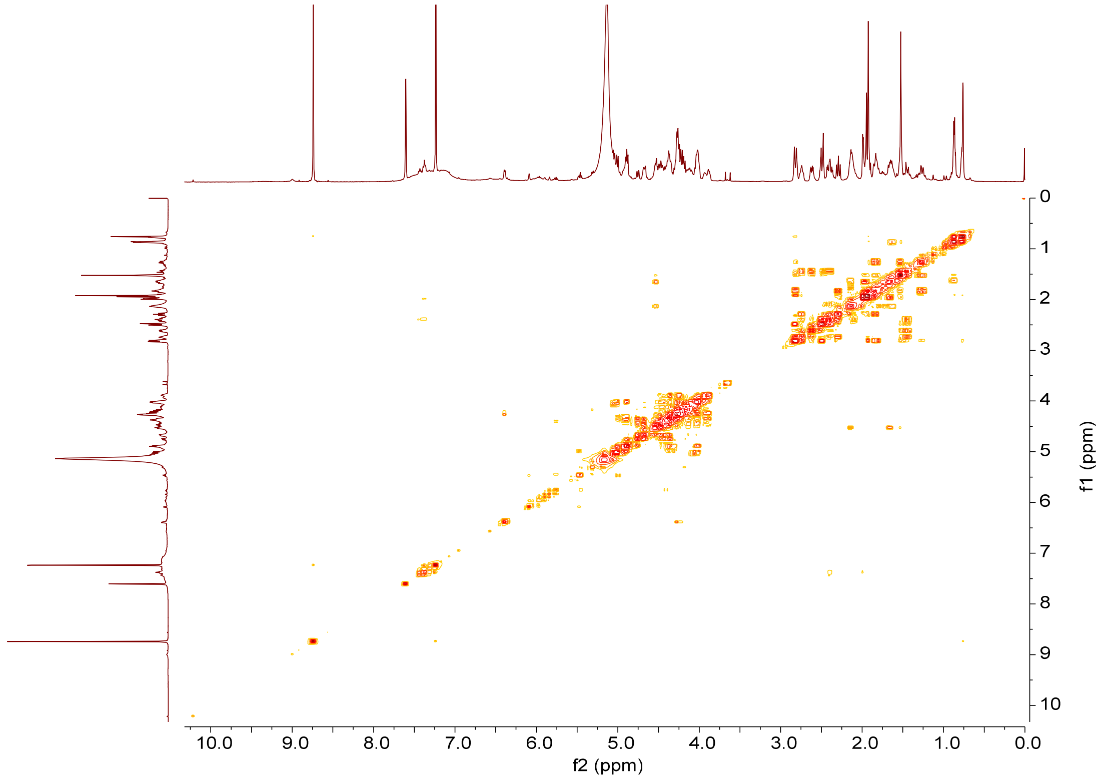

Figure S5. ^{1}H-^{1}H COSY spectrum of Siraitic acid III E (**4**).

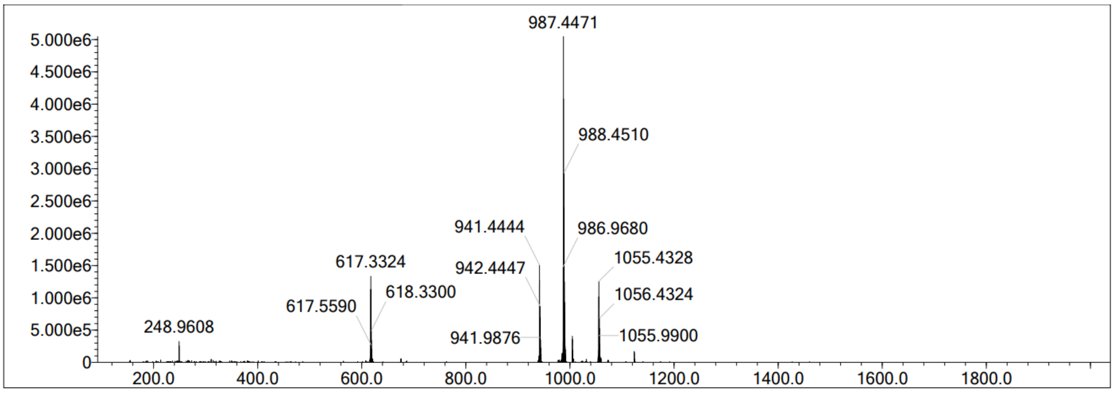

Figure S6. The HREIMS spectroscopic data of Siraitic acid III E (**4**).

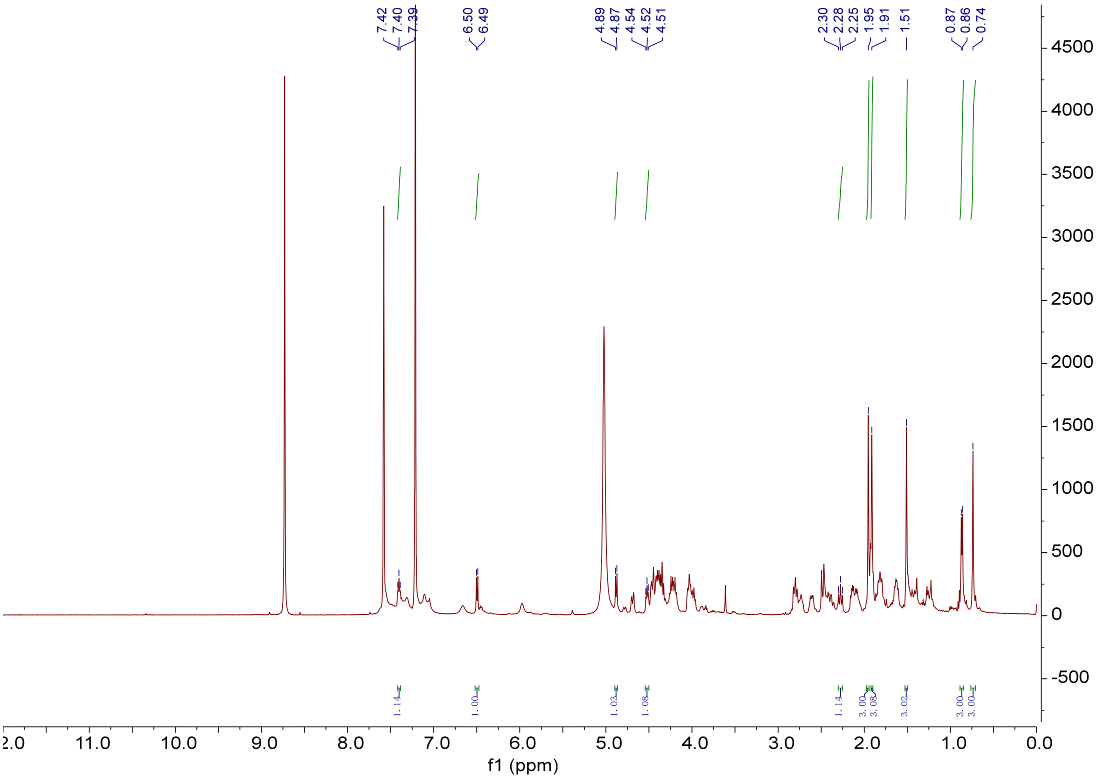

Figure S7. ^{1}H NMR spectrum of Siraitic acid IIb E (**6**) (500 MHz in pyridine-d5).

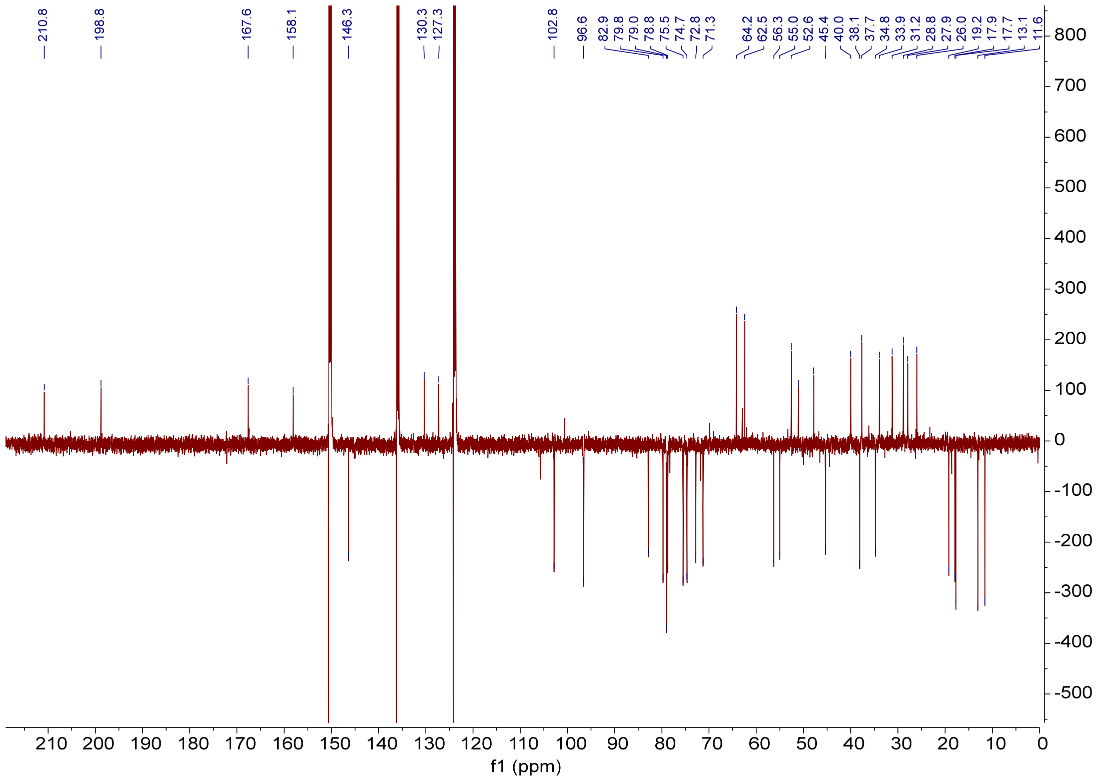

Figure S8. ^{13}C NMR spectrum of Siraitic acid IIb E (**6**) (125 MHz in pyridine-d5).

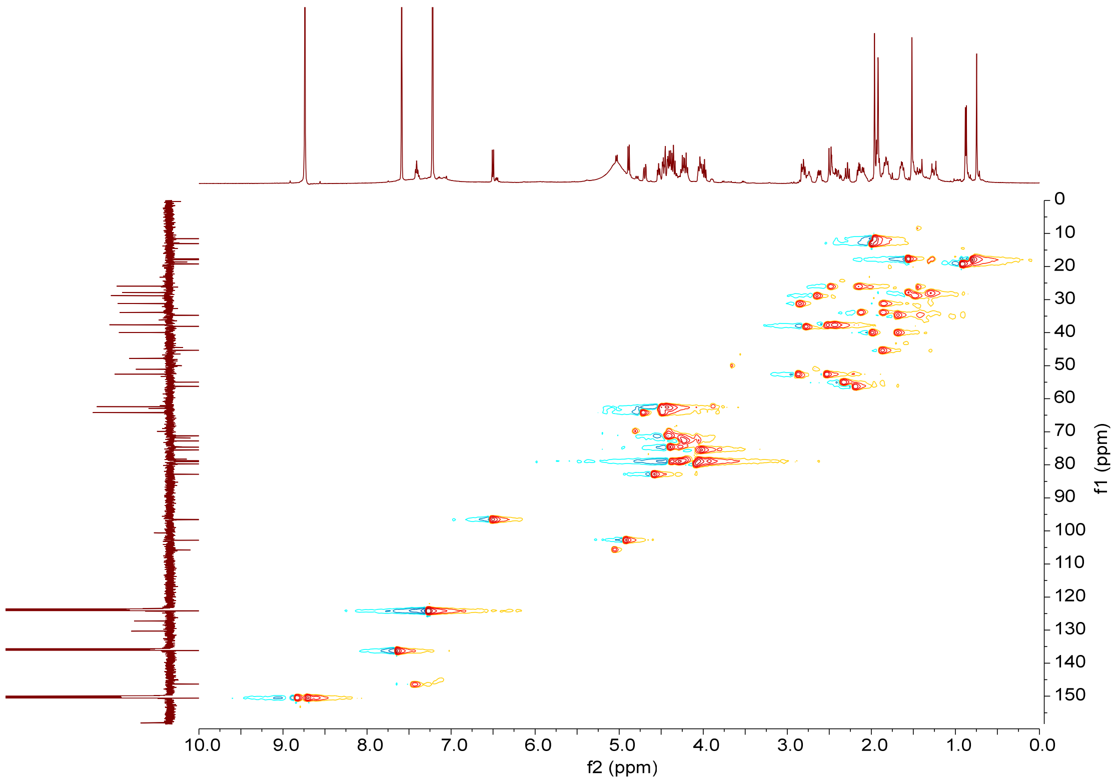

Figure S9. The HSQC spectrum of Siraitic acid IIb E (**6**).

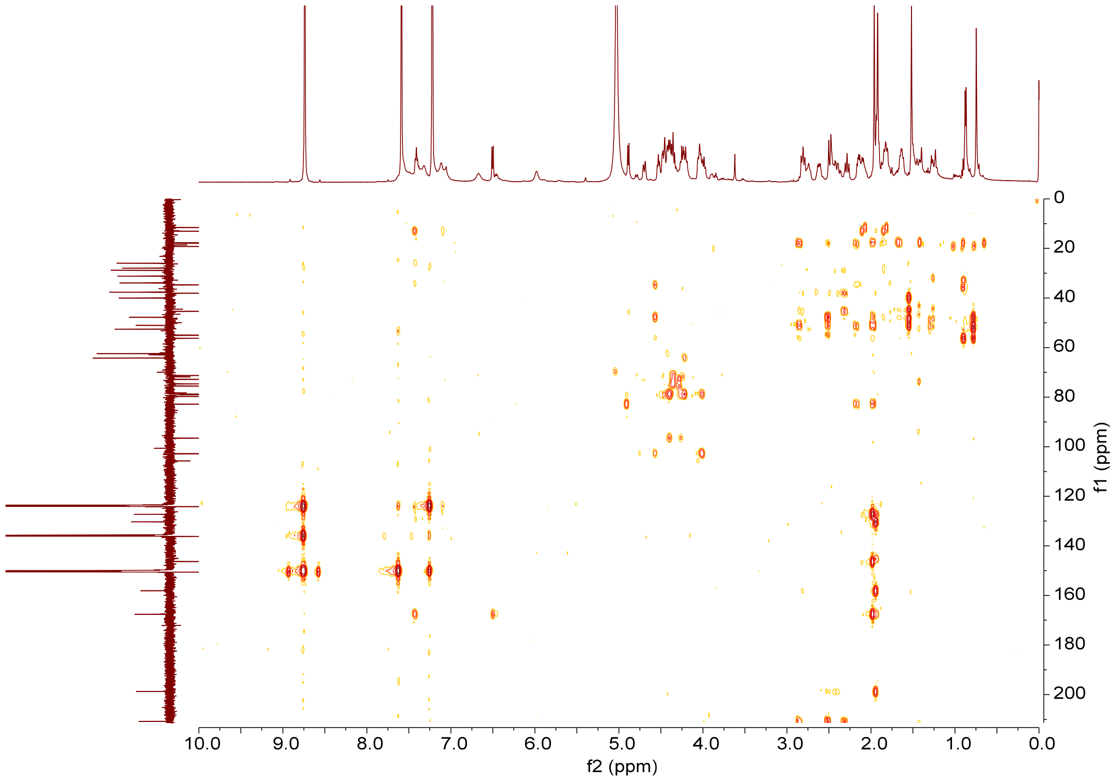

Figure S10. The HMBC spectrum of Siraitic acid IIb E (**6**).

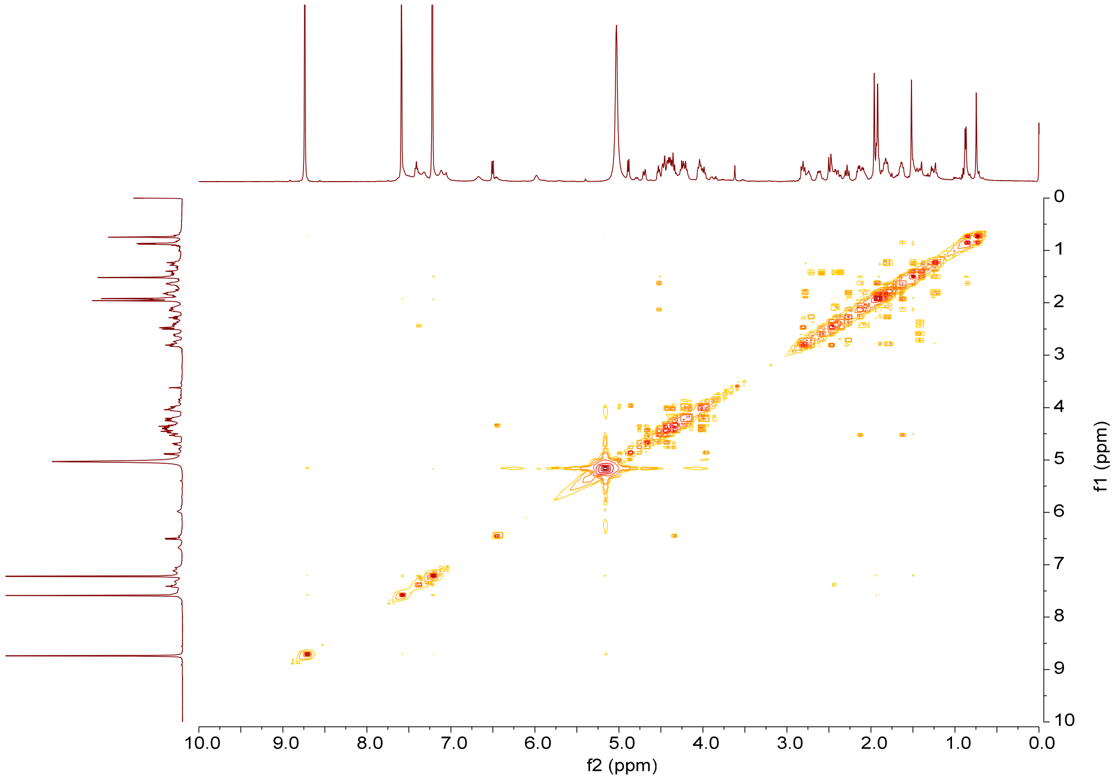

Figure S11. ^{1}H-^{1}H COSY spectrum of Siraitic acid IIb E (**6**).

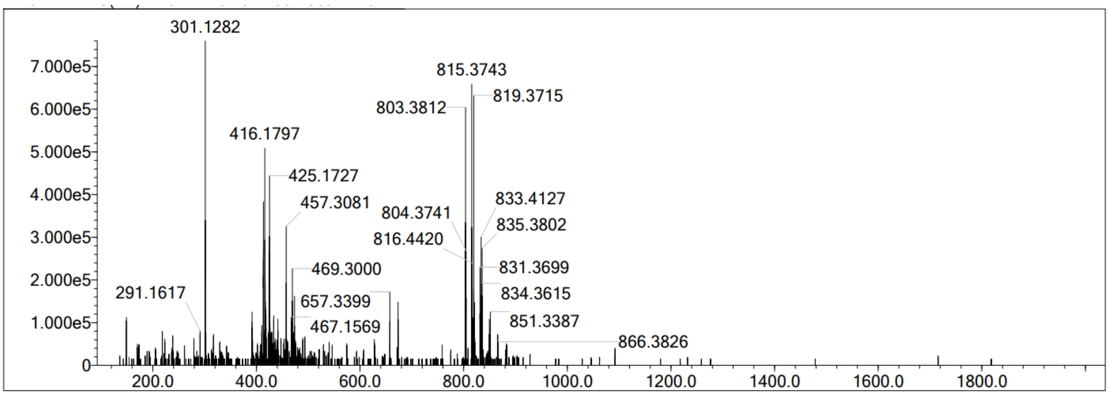

Figure S12. The HREIMS spectroscopic data of Siraitic acid IIb E (**6**).

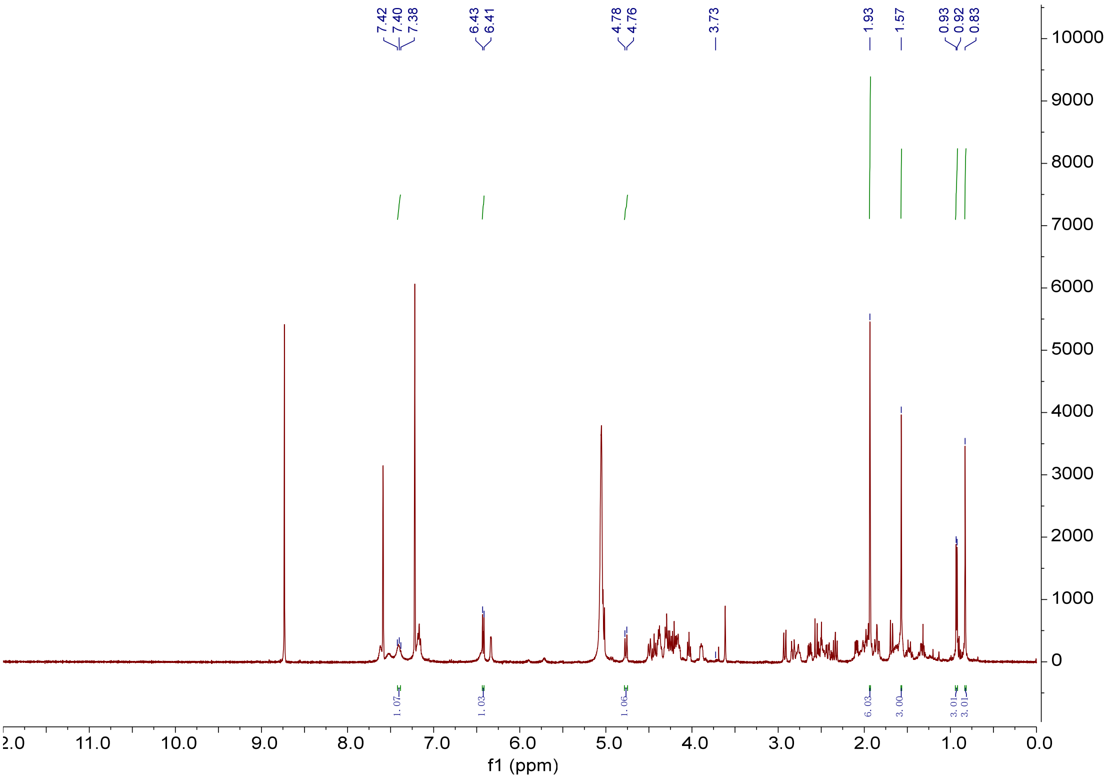

Figure S13. ^{1}H NMR spectrum of Siraitic acid II E (**7**) (500 MHz in pyridine-d5).

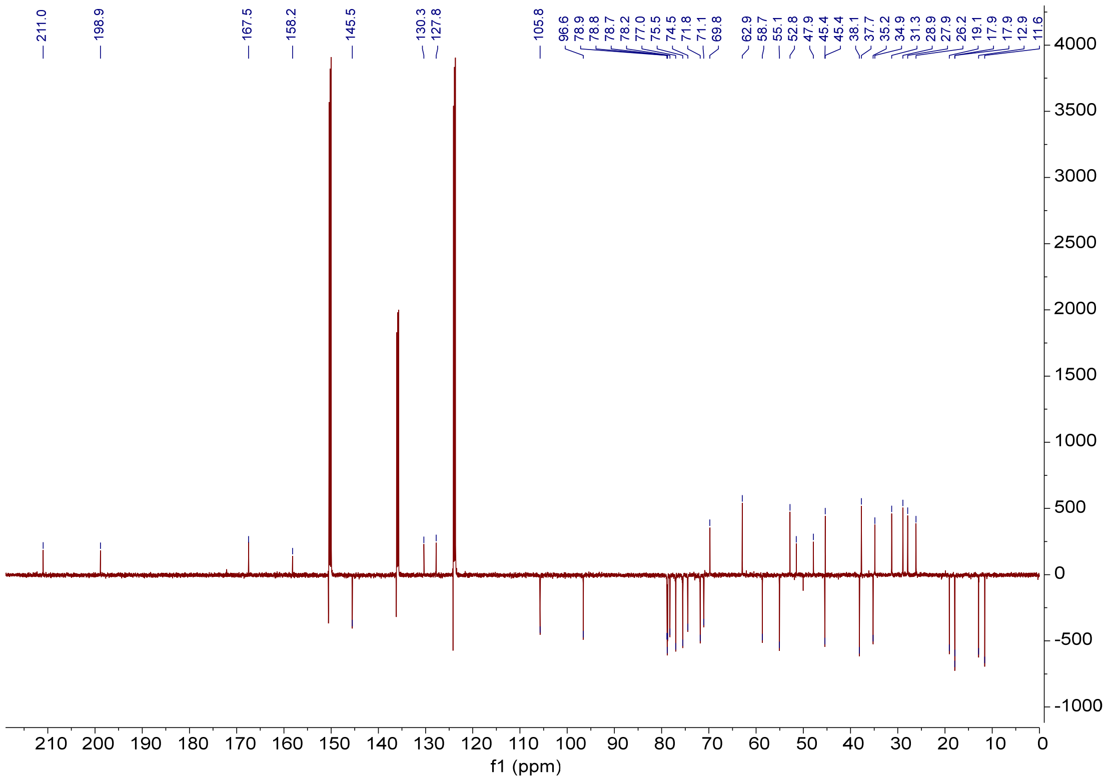

Figure S14. ^{13}C NMR spectrum of Siraitic acid II E (**7**) (125 MHz in pyridine-d5).

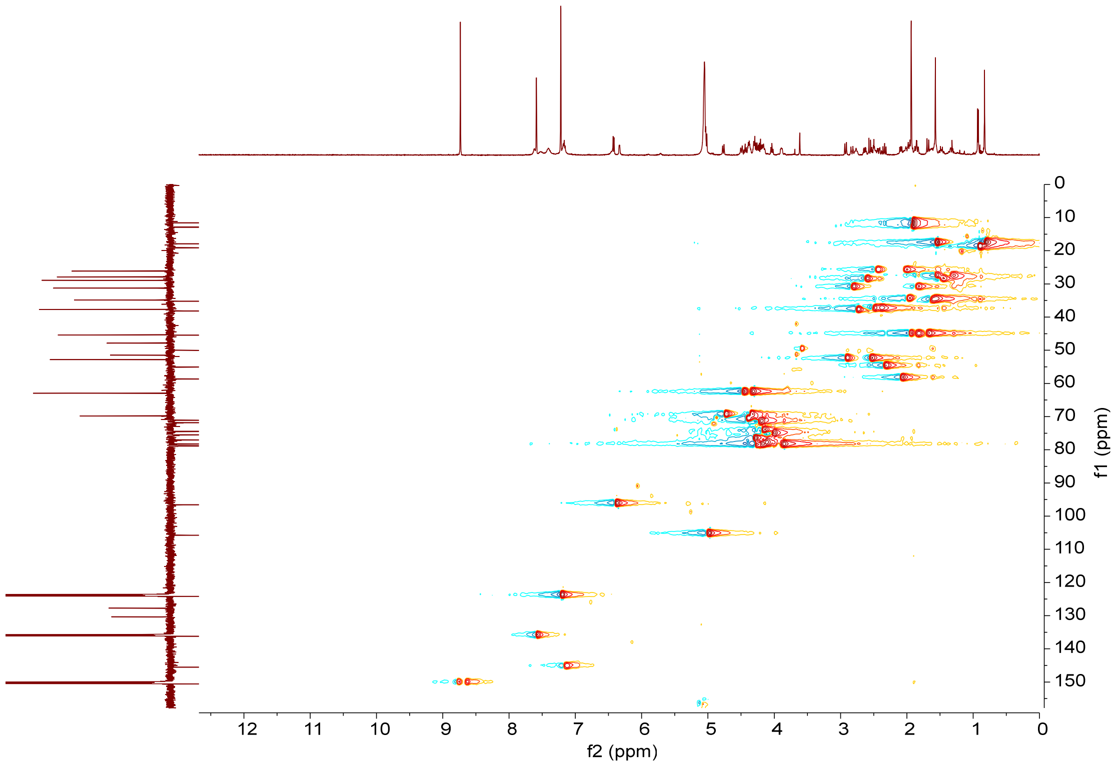

Figure S15 The HSQC spectrum of Siraitic acid II E (**7**).

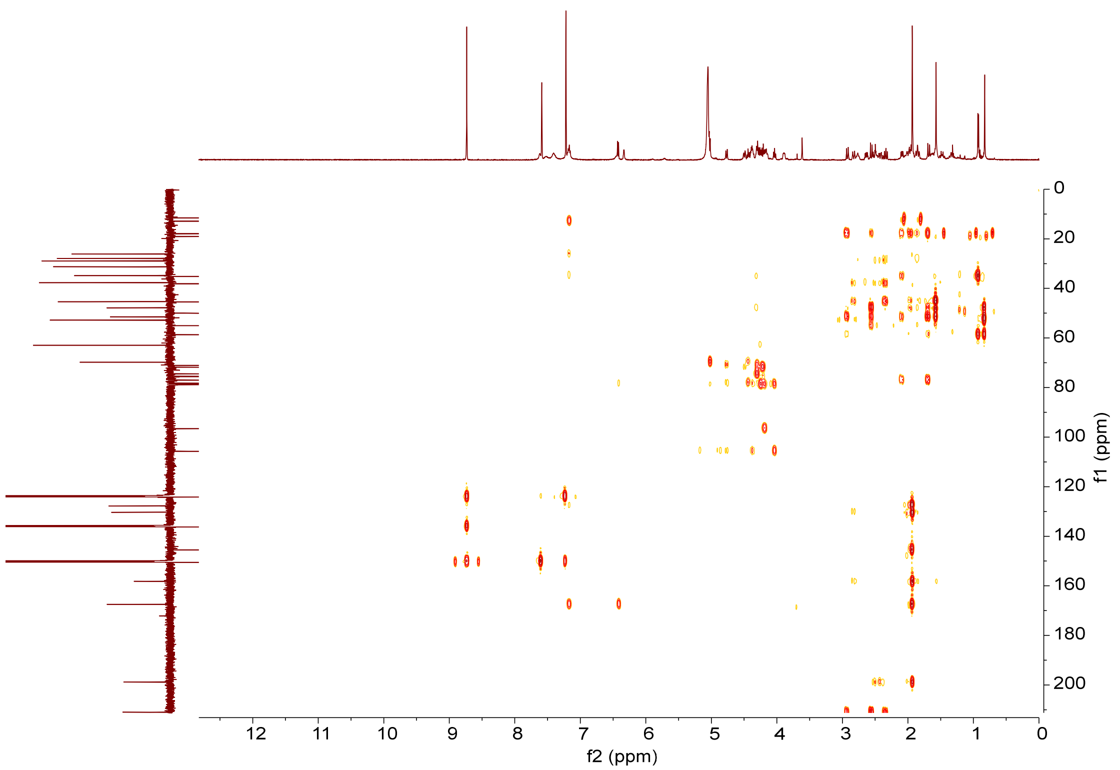

Figure S16. The HMBC spectrum of Siraitic acid II E (**7**).

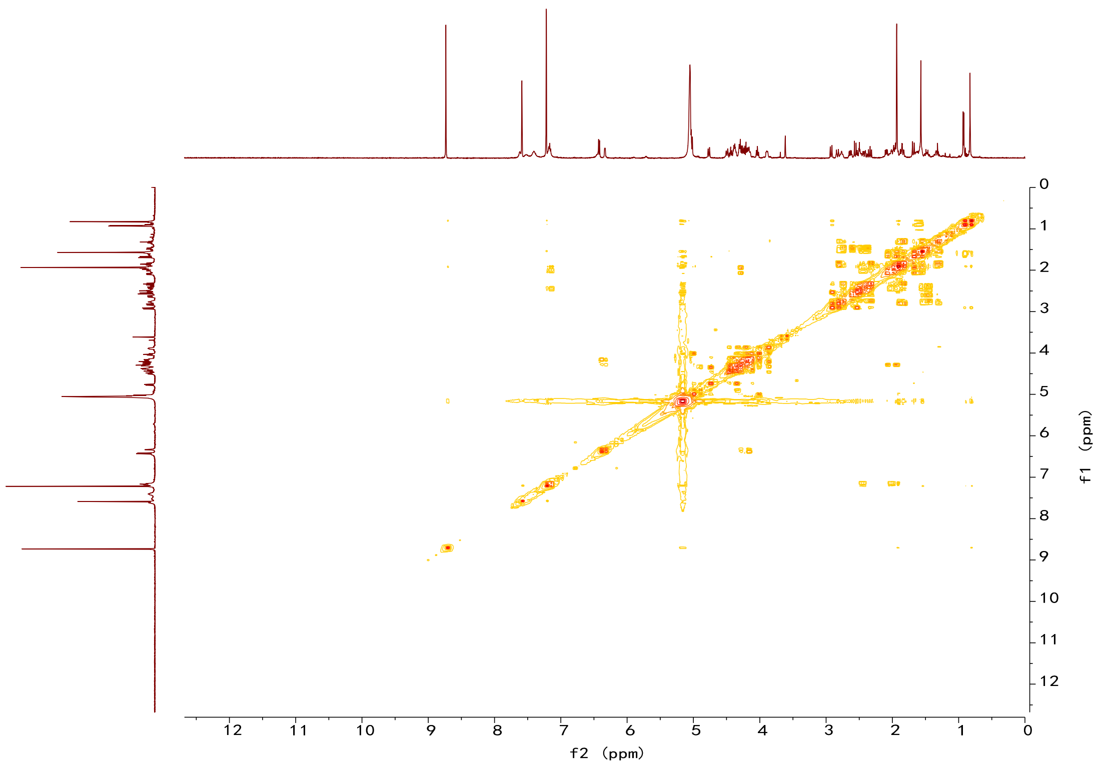

Figure S17. ^{1}H-^{1}H COSY spectrum of Siraitic acid II E (**7**).

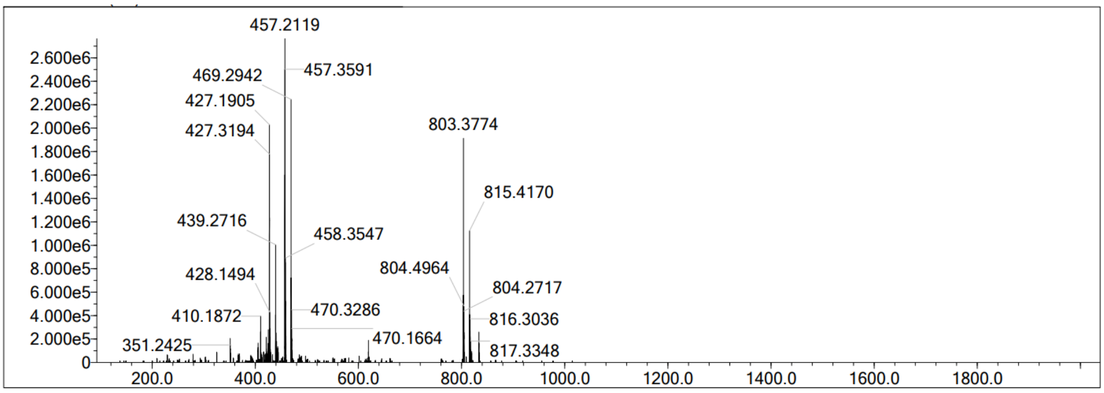

Figure S18. The HREIMS spectroscopic data of Siraitic acid II E (**7**).

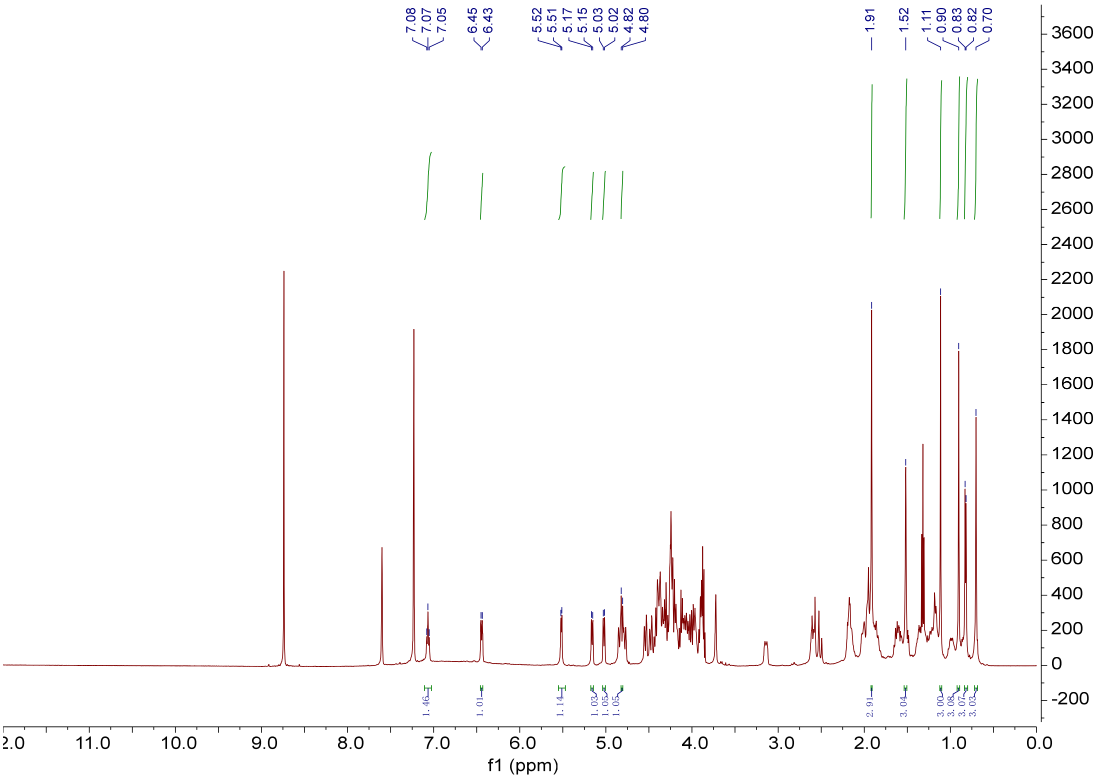

Figure S19. ^{1}H NMR spectrum of Siraitic acid IV H (**8**) (500 MHz in pyridine-d5).

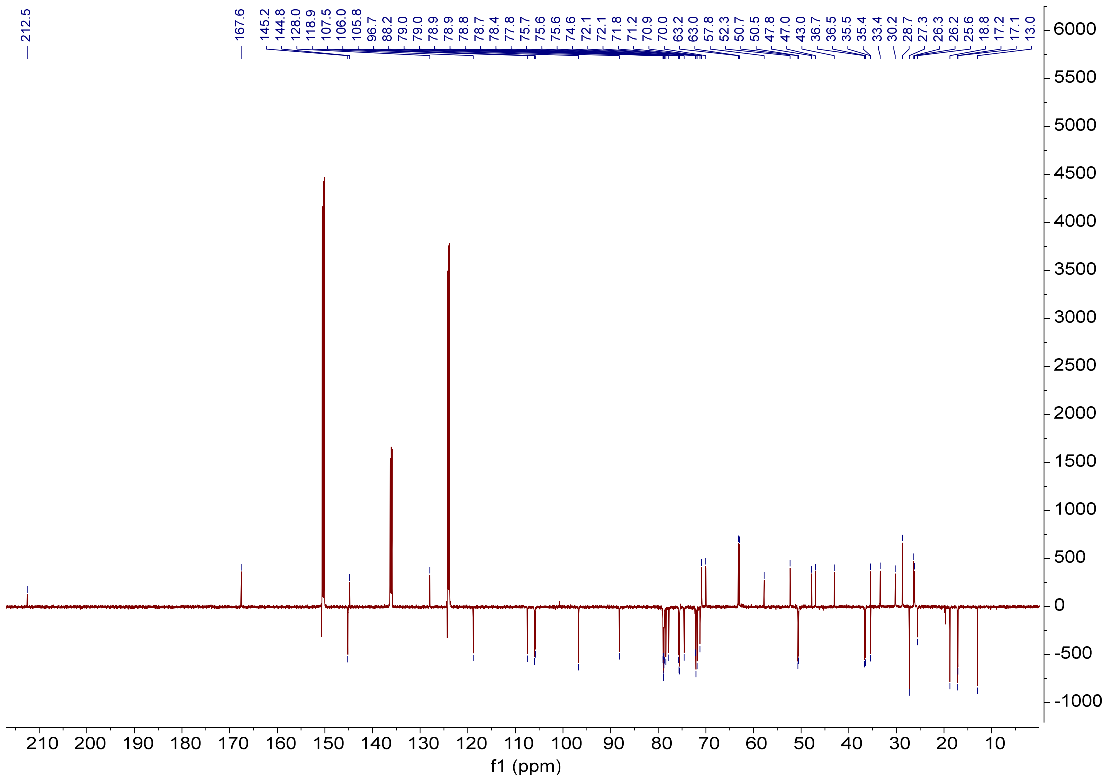

Figure S20. ^{13}C NMR spectrum of Siraitic acid IV H (**8**) (125 MHz in pyridine-d5).

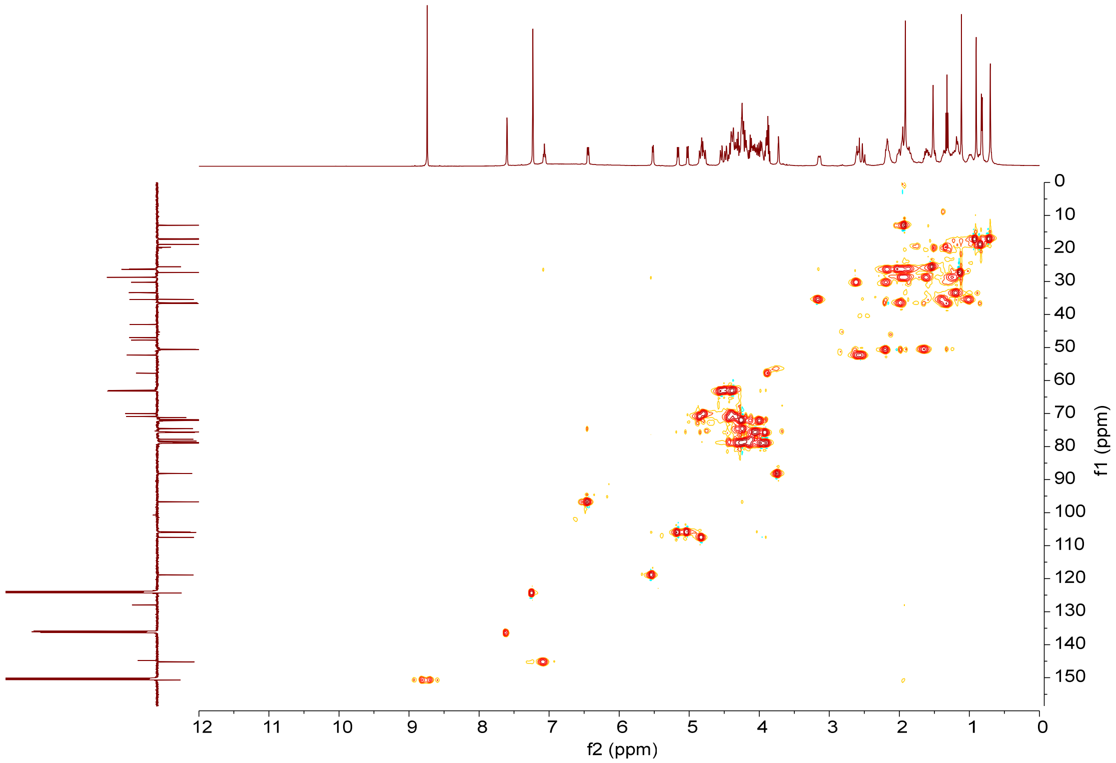

Figure S21 The HSQC spectrum of Siraitic acid IV H (**8**).

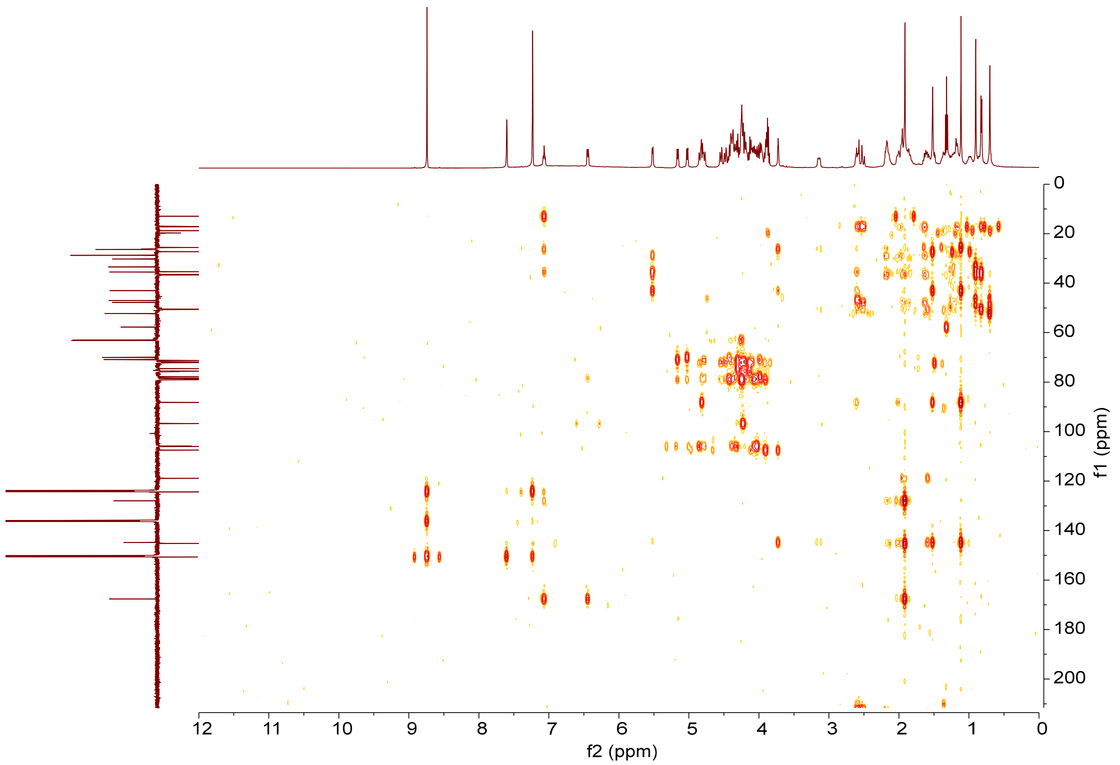

Figure S22. The HMBC spectrum of Siraitic acid IV H (**8**).

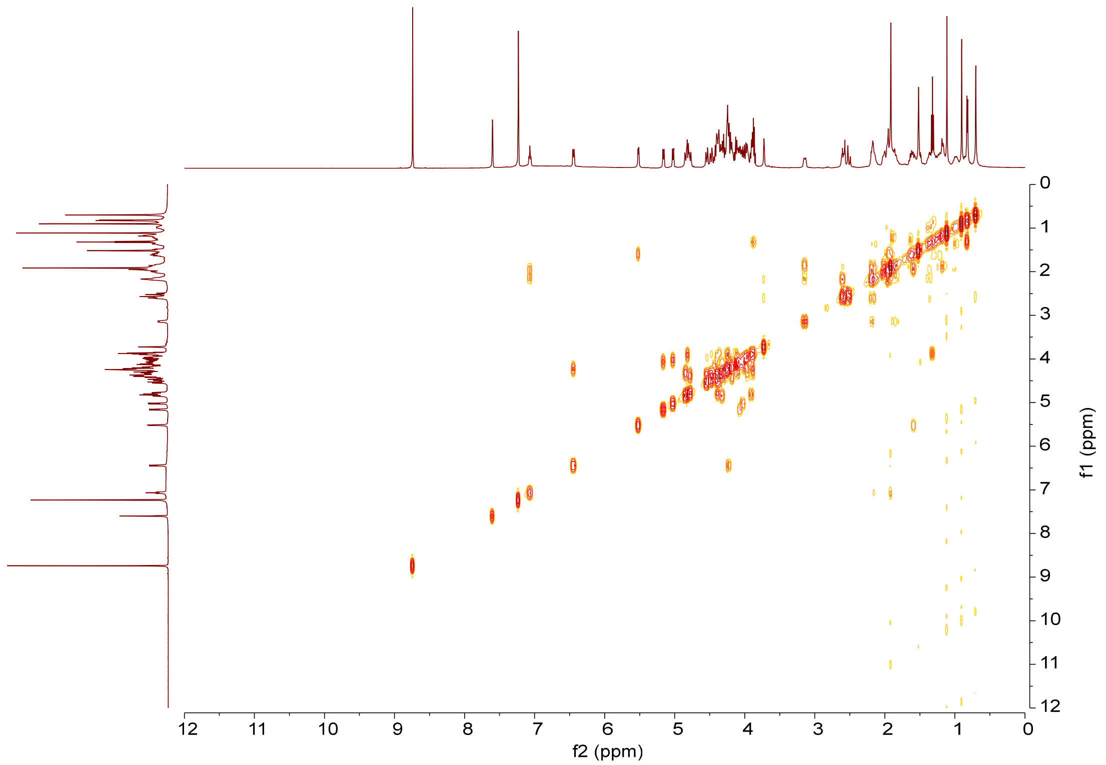

Figure S23. ^{1}H-^{1}H COSY spectrum of Siraitic acid IV H (**8**).

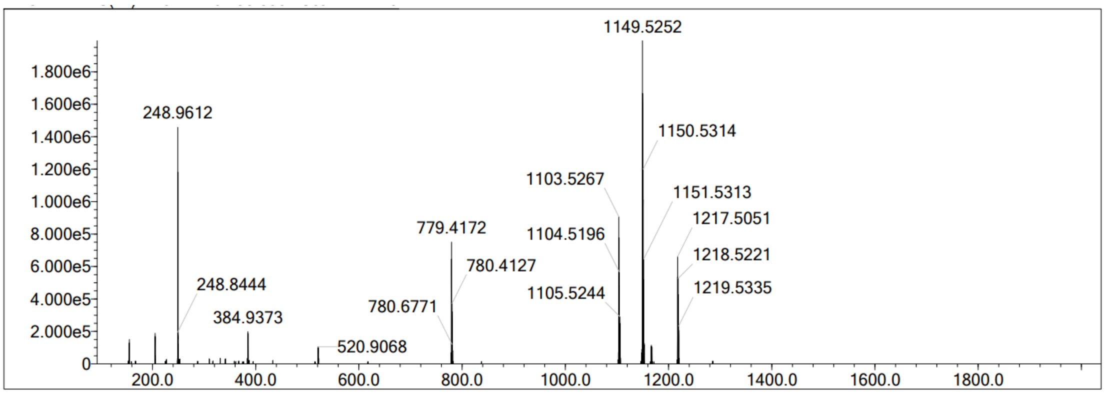

Figure S24. The HREIMS spectroscopic data of Siraitic acid IV H (**8**).

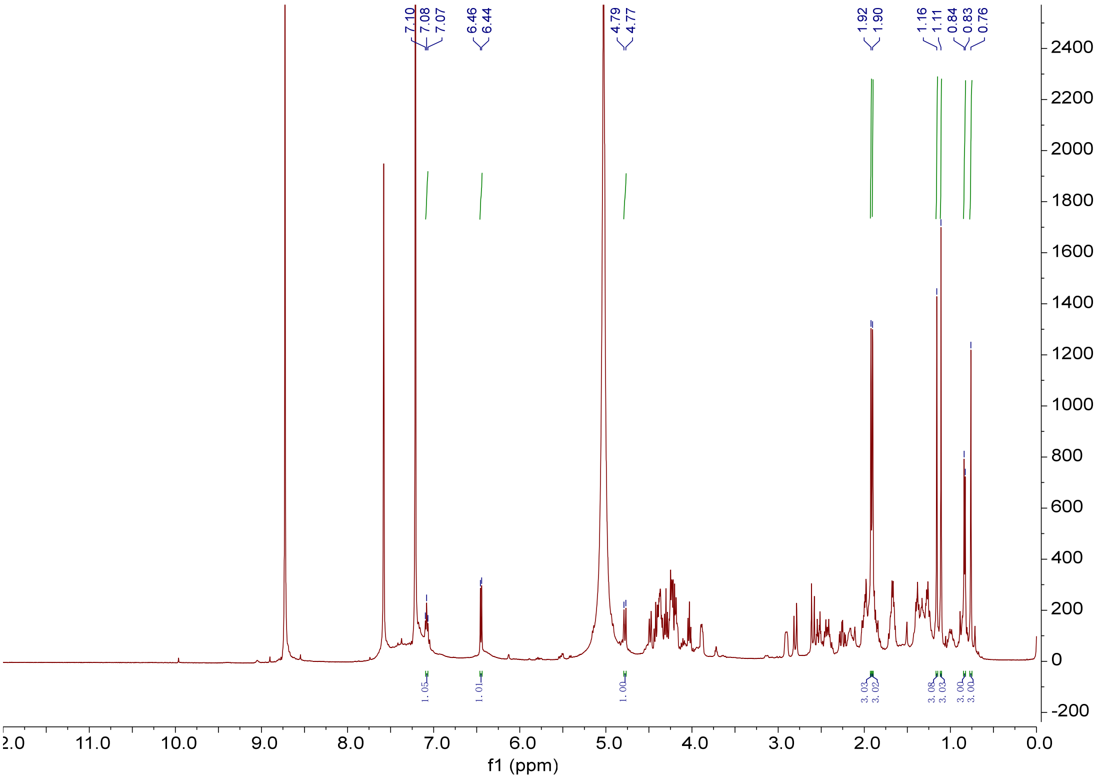

Figure S25. ^{1}H NMR spectrum of Siraitic acid II G (**9**) (500 MHz in pyridine-d5).

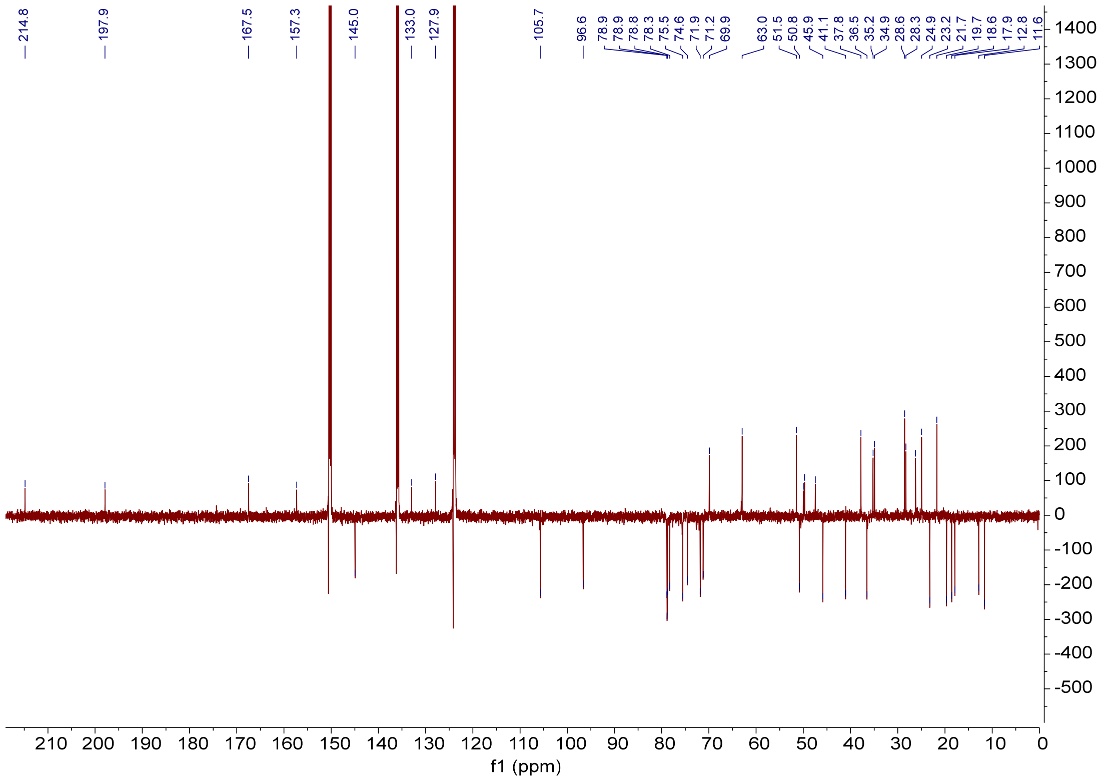

Figure S26. ^{13}C NMR spectrum of Siraitic acid II G (**9**) (125 MHz in pyridine-d5).

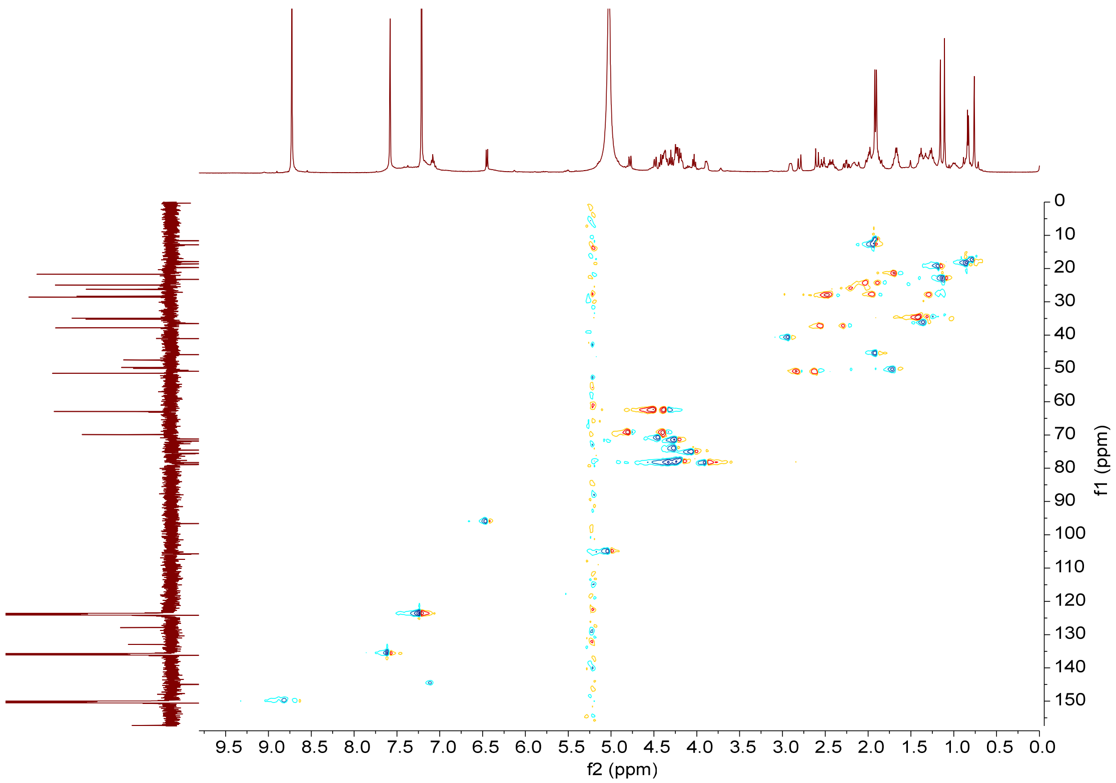

Figure S27 The HSQC spectrum of Siraitic acid II G (**9**).

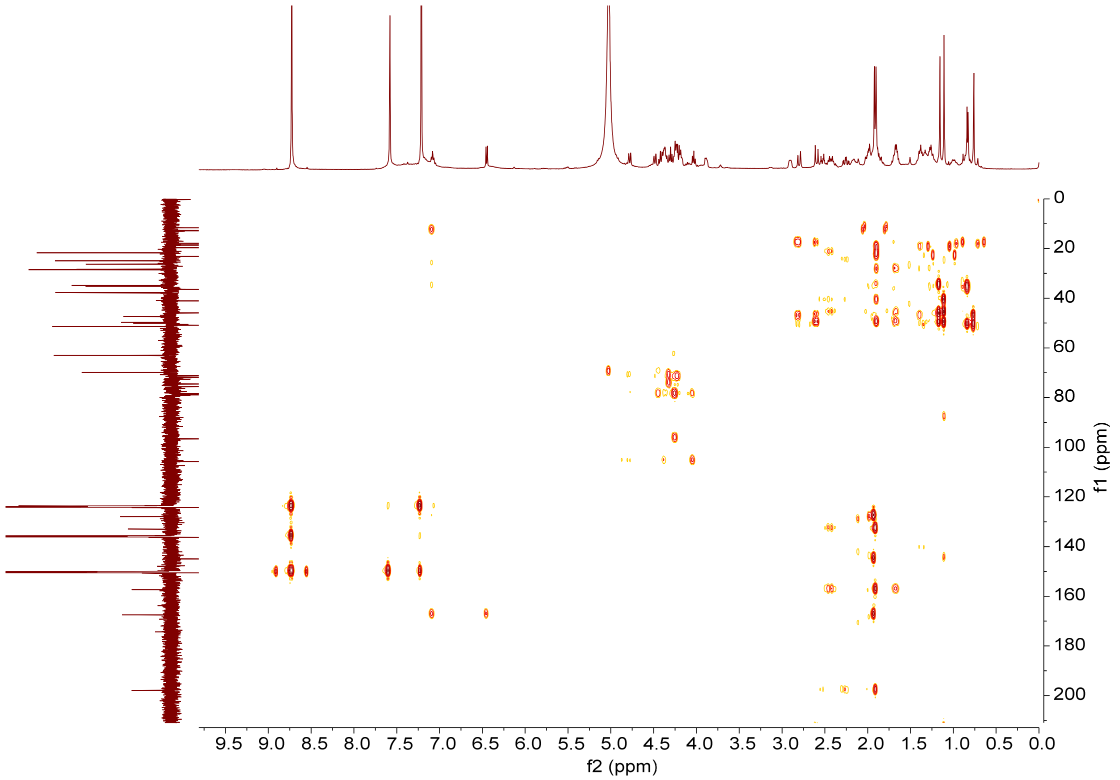

Figure S28. The HMBC spectrum of Siraitic acid II G (**9**).

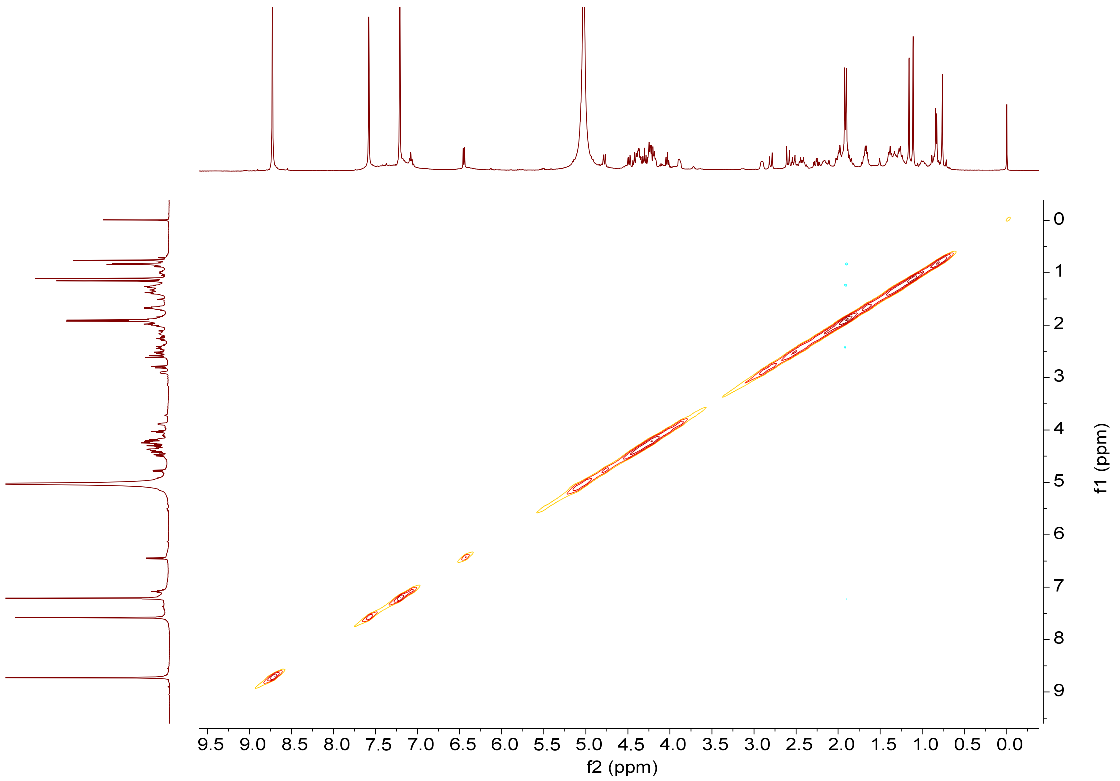

Figure S29. ^{1}H-^{1}H COSY spectrum of Siraitic acid II G (**9**).

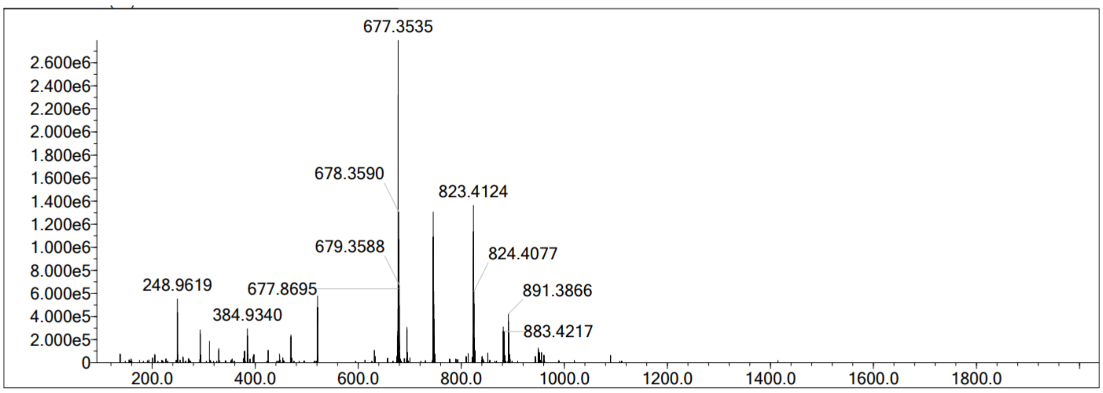

Figure S30. The HREIMS spectroscopic data of Siraitic acid II G (**9**).

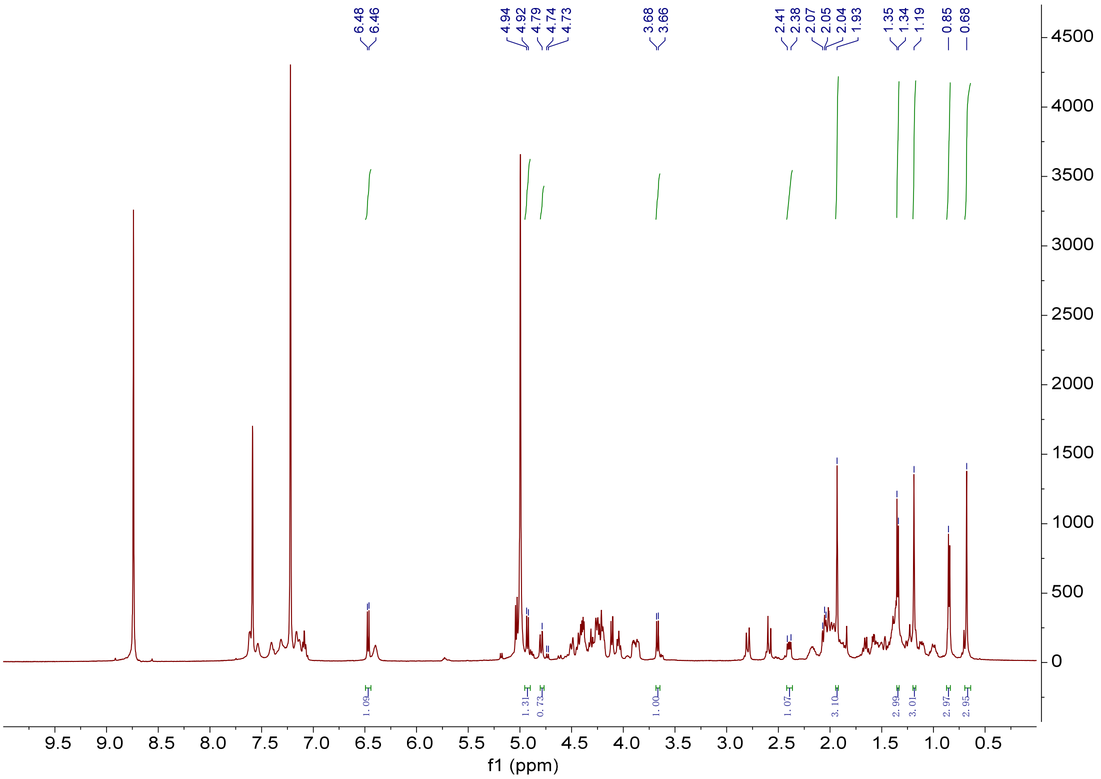

Figure S31. ^{1}H NMR spectrum of Siraitic acid II A (**11**) (500 MHz in pyridine-d5).

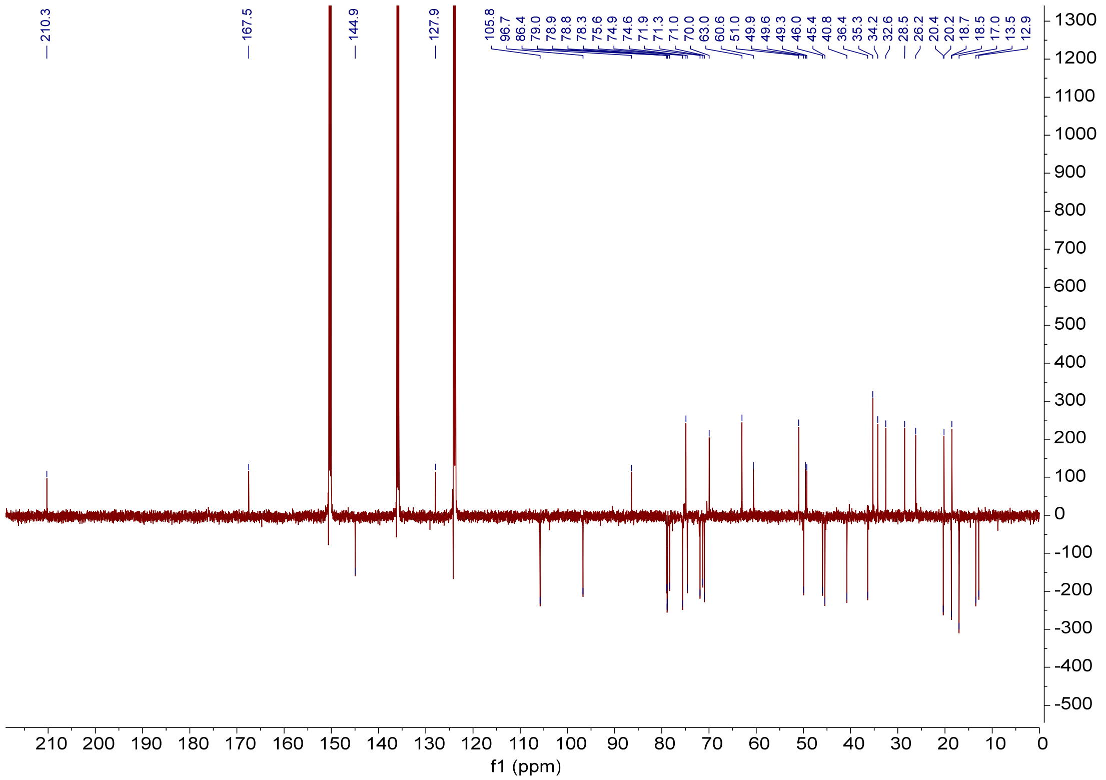

Figure S32. ^{13}C NMR spectrum of Siraitic acid II A (**11**) (125 MHz in pyridine-d5).

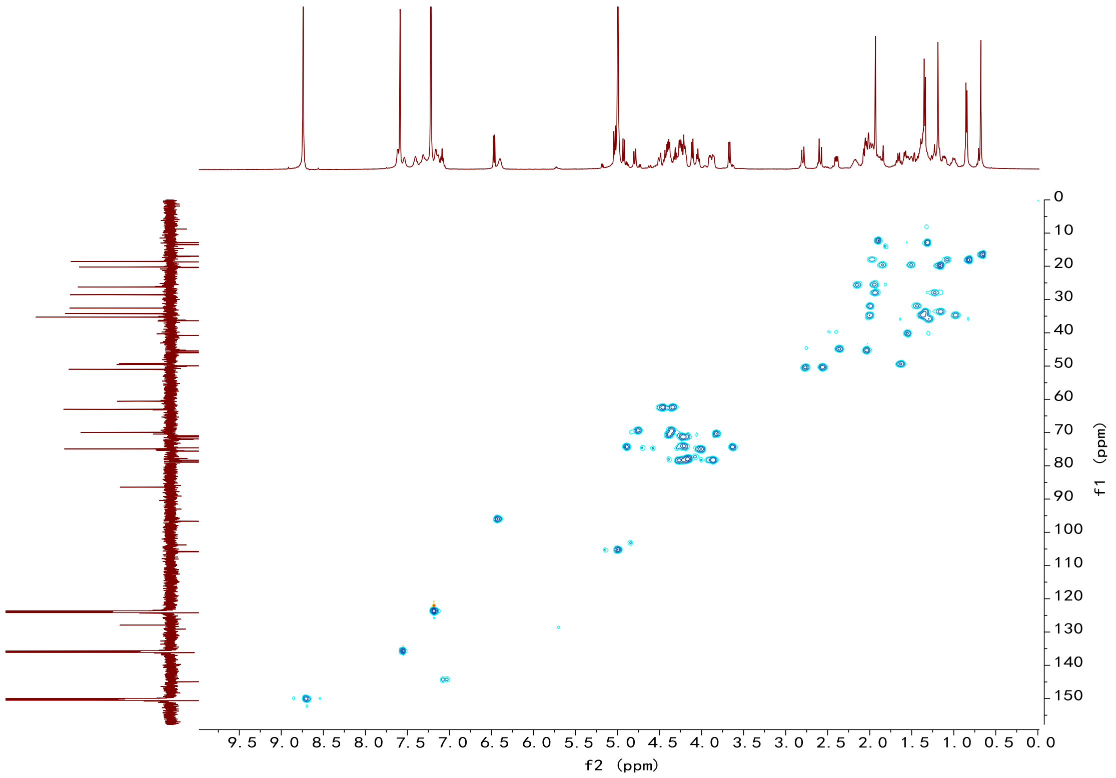

Figure S33 The HSQC spectrum of Siraitic acid II A (**11**).

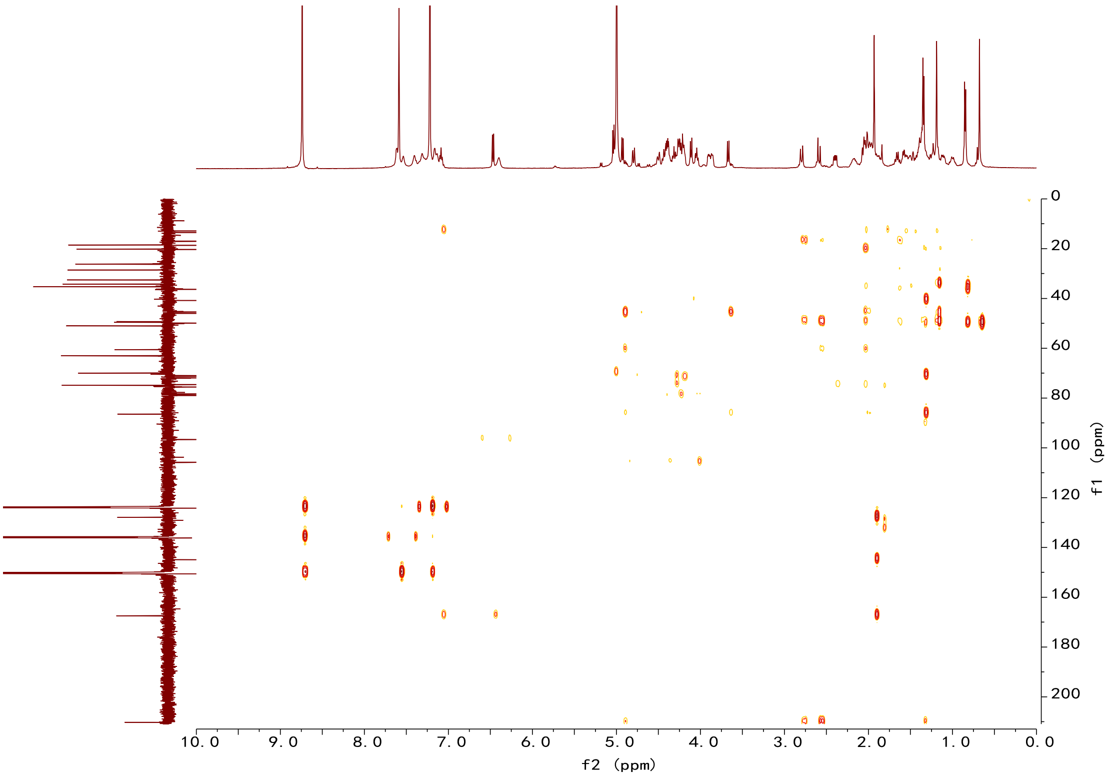

Figure S34. The HMBC spectrum of Siraitic acid II A (**11**).

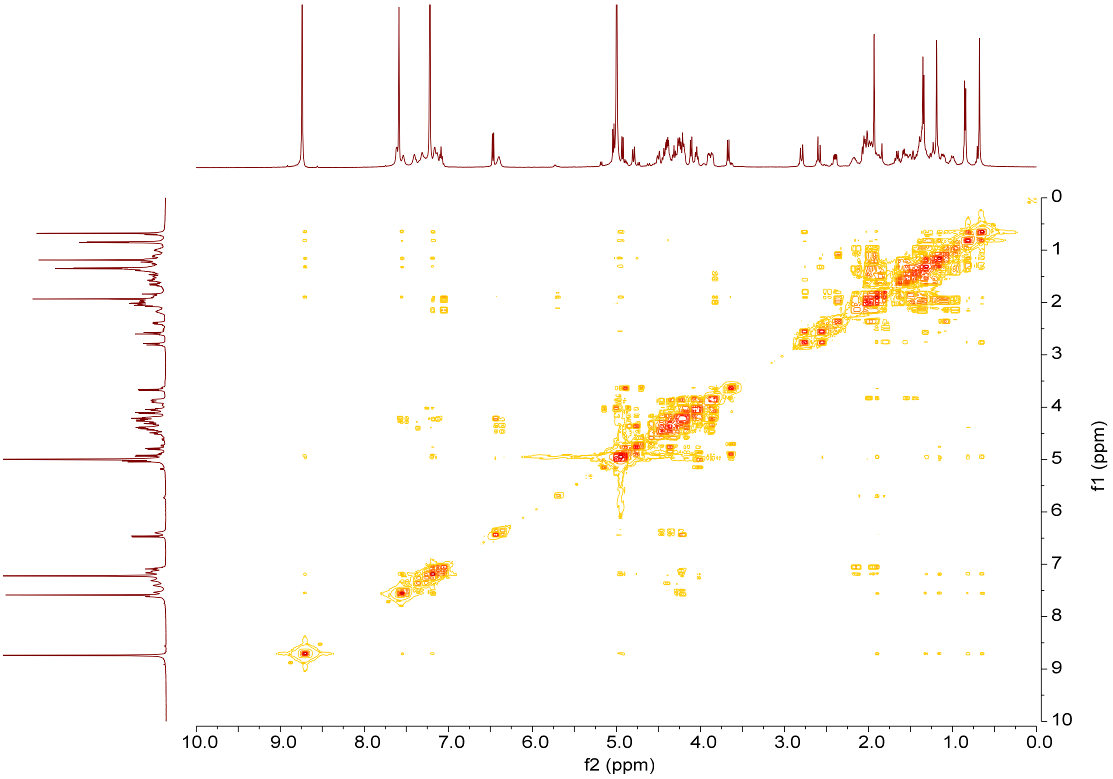

Figure S35. ^{1}H-^{1}H COSY spectrum of Siraitic acid II A (**11**).

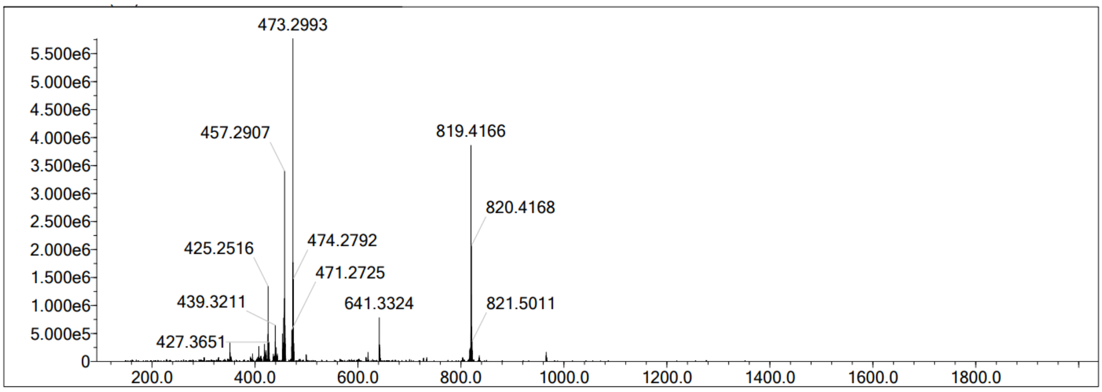

Figure S36. The HREIMS spectroscopic data of Siraitic acid II A (**11**).

NMR data for the known compounds 1-3, 5, 12-17were given as follows:

(-)-lariciresinol (**1**): ^{1}H-NMR δ: 7.34 (1H, d, J = 2.0 Hz, H-2), 7.28 (1H, d, J = 8.1 Hz, H-5'), 7.19 (1H, dd, J = 8.0, 2.0 Hz, H-6'), 7.01 (1H, d, J = 2.0 Hz,H-2'), 6.92 (1H, dd, J = 8.0, 2.0 Hz, H-6), 6.46 (1H, d, J = 8.1 Hz, H-5), 5.37 (1H, d, J = 6.0 Hz, H-7), 4.33 (1H, dd, J = 8.2, 6.7 Hz, H-9'a), 4.27 (1H, dd, J = 10.8, 6.9 Hz, H-9a), 4.15 (1H, dd, J = 10.8, 6.6 Hz, H-9b), 4.09 (1H, dd, J = 8.3, 6.9 Hz, H-9'b), 3.73 (3H, s, OCH_3), 3.72 (3H, s, OCH_3), 3.27 (1H, dd, J = 13.6, 5.0 Hz, H-7'a), 3.12 (1H, m, H-8'), 2.83 (1H, m, H-8), 2.80 (1H, dd, J = 13.6, 10.4 Hz, H-7'b). ^{13}C-NMR δ: 149.2 (C-3, C-4'), 147.9 (C-4), 147.0 (C-3'), 136.4 (C-1), 133.1 (C-1'), 122.3 (C-6'), 119.9 (C-6), 117.1 (C-5'), 116.8 (C-5), 113.7 (C-2'), 111.1 (C-2), 83.9 (C-7'), 73.7 (C-9'), 60.5 (C-9), 56.3 (OCH_3), 54.4 (C-8'), 43.8 (C-8), 33.9 (C-7).

3,4'-dimethoxy-4,9,9'-trihydroxy-benzofuranolignan-7'-ene (**2**): ^{1}H-NMR δ: 7.34 (1H, d, J = 2.8 Hz, H-2', 6'), 7.25 (1H, dd, J = 8.0, 2.3 Hz, H-6), 7.22 (1H, d, J = 8.0 Hz, H-5), 7.17 (1H, s, H-2), 6.94 (1H, d, J = 15.8 Hz, H-7'), 6.59 (1H, dt, J = 15.8, 5.3 Hz, H-8'), 6.11 (1H, d, J = 6.8 Hz, H-7), 4.62 (2H, d, J = 4.5 Hz, H-9'), 4.26 (1H, m, H-9), 3.99 (1H, m, H-8), 3.85 (3H, s, OCH_3), 3.65 (3H, s, OCH_3). ^{13}C-NMR δ: 149.4 (C-3), 149.3 (C-4), 148.7 (C-4'), 145.4 (C-3'), 134.1 (C-1), 132.3 (C-5'), 131.1 (C-1'), 130.4 (C-7'), 129.5 (C-8'), 120.3 (C-6), 117.0 (C-5), 115.4 (C-6'), 112.1 (C-2'), 111.3 (C-2), 89.2 (C-7), 64.7 (C-9), 63.5 (C-9'), 56.7 (OCH_3), 56.3 (OCH_3), 55.2 (C-8).

23,24-dihydrocucurbitacin F (**3**): ^{1}H-NMR δ 5.73 (1H, d, J = 5.0 Hz, H-6), 4.93 (1H, t, J = 6.0 Hz, H-16), 4.17 (1H, m, H-2), 3.46 (1H, d, J = 6.0 Hz, H-3), 1.58 (3H, s, H-21), 1.51 (3H, s, H-30), 1.47 (3H, s, H-19), 1.37 (6H, s, H-26, 27), 1.31 (3H, s, H-28), 1.23 (3H, s, H-29), 1.20 (3H, s, H-18). ^{13}C-NMR δ 216.5(C-22), 213.6(C-11), 142.9(C-5), 119.2(C-6), 81.9(C-3), 80.6(C-20), 71.5 (C-2), 70.8 (C-16), 69.5 (C-25), 59.2 (C-17), 51.6 (C-14), 49.8 (C-12), 49.3 (C-13), 49.1 (C-9), 46.9 (C-15), 43.6 (C-8), 43.3 (C-4), 39.0 (C-24), 35.2 (C-10), 34.9 (C-1), 33.2 (C-23), 30.6 (C-26), 30.3 (C-27), 26.0 (C-30), 25.9(C-21), 24.6 (C-7), 23.0 (C-29), 21.0 (C-18), 20.8 (C-19), 19.6 (C-28).

Siraitic glycoside II F (**5**): ^{1}H-NMR δ 7.08 (1H, t, J = 7.5 Hz, H-24), 6.45 (1H, d,

J = 8.1 Hz, H-G_{II}1), 5.02 (1H, d, *J* = 7.9 Hz, H-G_{I}1), 4.60 (1H, s, H-19), 3.70 (1H, t, *J* = 9.9 Hz, H-8), 3.40 (1H, t, *J* = 8.4 Hz, H-10), 3.20 (1H, d, *J* =5.4 Hz, H-6), 1.94 (3H, s, H-26), 1.87 (3H, s, H-28), 1.22 (3H, s, H-30), 0.96 (3H, s, H-18), 0.90 (3H, d, *J* = 6.5 Hz, H-21). ^{13}C-NMR δ: 211.3 (C-11), 198.1 (C-3), 167.5 (C-27), 165.0 (C-5), 145.0 (C-24), 127.9 (C-25), 123.3 (C-4), 105.7 (C-G_{II}1), 96.6 (C-G_{I}1), 78.8 (C-G_{I}3), 78.7 (C-G_{II}3), 78.6 (C-G_{II}5), 78.2 (C-G_{I}5), 76.5 (C-19), 75.4 (C-G_{II}2), 74.4 (C-G_{I}2), 71.7 (C-G_{II}4), 71.1 (C-G_{I}4), 69.9 (C-G_{I}6), 62.8 (C-G_{II}6), 62.4 (C-9), 52.1 (C-14), 50.9 (C-12), 49.4 (C-17), 48.6 (C-6), 47.5 (C-13), 41.7 (C-10), 41.0 (C-8), 39.1 (C-2), 36.6 (C-20), 35.4 (C-22), 34.0 (C-15), 29.6 (C-16), 27.1 (C-1), 26.2 (C-23), 24.6 (C-7), 19.3 (C-30), 18.7 (C-21), 17.3 (C-18), 12.8 (C-26), 11.9 (C-28).

Siraitic acid II B (**12**): ^{1}H-NMR δ 7.09 (1H, t, *J* = 7.0 Hz, H-24), 6.47 (1H, d, *J* = 8.0 Hz, H-G_{II}1), 4.80 (1H, d, *J* = 9.1 Hz, H-G_{I}1), 4.74 (1H, d, *J* = 8.4 Hz, H-19), 3.66 (1H, d, *J* = 8.4 Hz, H-19), 1.93 (3H, s, H-28), 1.36 (3H, d, *J* =6.8 Hz, H-26), 1.25 (3H, s, H-30), 0.84 (3H, d, *J* = 6.4 Hz, H-21), 0.68 (3H, s, H-18). ^{13}C-NMR δ 210.2 (C-3), 210.2 (C-11), 167.6 (C-27), 145.0 (C-24), 128.0 (C-25), 105.9 (C- G_{II}1), 96.8 (C-G_{I}1), 90.6 (C-5), 79.0 (C-G_{II}3), 79.0 (C-G_{II}5), 78.9 (C-G_{I}5), 78.4 (C-G_{I}3), 75.7 (C-G_{I}2), 75.3 (C-19), 74.7 (C-G_{II}2), 72.0 (C-G_{II}4), 71.4 (C-G_{I}4), 70.0 (C-G_{I}6), 63.1 (C-G_{II}6), 60.4 (C-9), 51.1 (C-12), 50.8 (C-4), 50.1 (C-17), 49.6 (C-14), 49.2 (C-13), 46.1 (C-8), 45.3 (C-10), 40.4 (C-2), 36.4 (C-20), 36.3 (C-15), 35.4 (C-22), 34.3 (C-16), 28.6 (C-7), 26.3 (C-23), 26.1(C-6), 20.4 (C-1, 30), 20.4 (C-30), 18.7 (C-21), 17.1 (C-18), 12.9 (C-26), 8.9 (C-28).

23,24-dihydrocucurbitacin F-25-acetate (**13**): ^{1}H-NMR δ: 5.75 (1H, d, *J* = 4.3 Hz, H-6), 4.12 (1H, ddd, *J* = 11.4, 9.0, 4.1 Hz, H-2), 3.46 (1H, d, *J* = 9.1 Hz, H-3), 2.74 (1H, d, J = 12.9 Hz, H-10), 2.45 (2H, ddd, *J* = 12.3, 3.1, 4.0 Hz, H-1), 1.92 (3H, s, $COCH_3$), 1.63 (3H, s, H-30), 1.56 (3H, s, H-27), 1.52 (3H, s, H-26), 1.50 (3H, s, H-28), 1.50 (3H, s, H-29), 1.32 (3H, s, H-19), 1.27 (3H, s, H-21), 1.25 (3H, s, H-18). ^{13}C-NMR δ: 35.1 (C-1), 71.5 (C-2), 81.9 (C-3), 43.3 (C-4), 142.9 (C-5), 119.2 (C-6), 24.6 (C-7), 34.9 (C-8), 49.2 (C-9), 43.6 (C-10), 213.6 (C-11), 49.7 (C-12), 49.3 (C-13), 51.6 (C-14), 46.9 (C-15), 70.9 (C-16), 59.5 (C-17), 20.9 (C-18), 20.8 (C-19), 82.1 (C-20), 26.0 (C-21), 215.6 (C-22), 32.7 (C-23), 35.9 (C-24), 80.7 (C-25), 26.4 (C-26), 26.0 (C-27), 26.5 (C-28), 22.7 (C-29), 19.7 (C-30), 170.6 ($COCH_3$), 22.9 ($COCH_3$).

Siraitic acid II C(**14**): ^{1}H-NMR δ 3.66 (1H, d, J =8.4 Hz, H-19a), 4.74 (1H, d, J = 8.4 Hz, H-19b), 0.84 (3H, d, J = 6.4 Hz, H-21), 0.68 (3H, s, H-18), 1.36 (3H, d, J = 6.8 Hz, H-26), 7.09 (1H, t, J = 7.0 Hz, H-24), 1.93 (3H, s, H-28), 1.25 (3H, s, H-30), 4.80 (1H, d, J = 9.1 Hz, H-G_{I}1), 6.47 (1H, d, J = 8.0 Hz, H-G_{II}1); ^{13}C-NMR δ: 29.0 (C-1), 37.8 (C-2), 199.1 (C-3), 158.4 (C-4), 130.5 (C-5), 29.1(C-6), 28.0 (C-7), 45.3 (C-8), 55.1 (C-9), 38.2 (C-10), 211.5 (C-11), 52.8 (C-12), 49.0 (C-13), 50.8 (C-14), 32.5 (C-15), 31.4 (C-16), 50.1 (C-17), 16.6 (C-18), 36.6 (C-20), 18.6 (C-21), 35.3 (C-22), 26.3 (C-23), 145.1 (C-24), 128.0 (C-25), 13.0 (C-26), 167.6 (C-27), 11.8 (C-28), 17.6 (C-30), 96.8 (C-G_{I}1), 74.7 (C-G_{I}2), 78.9 (C-G_{I}3), 71.3 (C-G_{I}4), 78.4 (C-G_{I}5), 63.1 (C-G_{I}6), 105.8 (C- G_{II}1), 72.0 (C-G_{II}2), 79.0 (C-G_{II}3), 70.0 (C-G_{II}4), 75.7 (C-G_{II}5), 57.9 (C-G_{II}6).

cucurbitacin B (**15**): ^{1}H-NMR δ 2.68 (2H, m, H-1), 5.70 (1H, d, J = 4.3 Hz, H-6), 2.93 (1H, d, J = 14.6 Hz, H-10), 1.14 (3H, s, H-18), 1.22 (3H, s, H-19), 1.73 (3H, s, H-21), 7.38 (1H, d, J = 15.8 Hz, H-23), 7.42 (1H, d, J = 15.8 Hz, H-24), 1.54 (3H, s, H-26), 1.57 (3H, s, H-27), 1.46 (3H, s, H-28), 1.30 (3H, s, H-29), 1.60 (3H, s, H-30), 1.92 (3H, s, $COCH_3$); ^{13}C-NMR δ: 37.4 (C-1), 72.9 (C-2), 213.7 (C-3), 51.4 (C-4), 141.8 (C-5), 120.7 (C-6), 24.7 (C-7), 43.4 (C-8), 49.3 (C-9), 34.8 (C-10), 213.5 (C-11), 49.6 (C-12), 49.1 (C-13), 51.5 (C-14), 46.8 (C-15), 71.2 (C-16), 60.4 (C-17), 21.1 (C-18), 20.5 (C-19), 80.3 (C-20), 26.1 (C-21), 204.9 (C-22), 123.0 (C-23), 150.5 (C-24), 80.3 (C-25), 26.7 (C-26), 27.0 (C-27), 29.9 (C-28), 22.2 (C-29), 19.4 (C-30), 170.3 ($COCH_3$), 22.3 ($COCH_3$).

23,24-dihydrocucurbitacin B (**16**): ^{1}H-NMR δ 2.70 (2H, m, H-1), 5.71 (1H, d, J = 4.7 Hz, H-6), 2.90 (1H, d, J = 14.5 Hz, H-10), 1.14 (3H, s, H-18), 1.24 (3H, s, H-19), 1.58 (3H, s, H-21), 1.50 (3H, s, H-26), 1.52 (3H, s, H-27), 1.46 (3H, s, H-28), 1.32 (3H, s, H-29), 1.63 (3H, s, H-30), 1.93 (3H, s, $COCH_3$); ^{13}C-NMR δ: 37.4 (C-1), 72.9 (C-2), 213.7 (C-3), 51.4 (C-4), 141.8 (C-5), 120.7 (C-6), 24.6 (C-7), 43.3 (C-8), 49.2 (C-9), 34.7 (C-10), 213.3 (C-11), 49.2 (C-12), 49.8 (C-13), 51.5 (C-14), 46.8 (C-15), 70.8 (C-16), 59.6 (C-17), 20.5 (C-18), 20.8 (C-19), 80.6 (C-20), 26.0 (C-21), 215.6 (C-22), 32.7 (C-23), 35.9 (C-24), 82.1 (C-25), 26.4 (C-26), 26.5 (C-27), 29.9 (C-28), 22.3 (C-29), 19.4 (C-30), 170.6 ($COCH_3$), 22.7 ($COCH_3$).

Dihydroisocucurbitacin B-25-acetate (**17**): ^{1}H-NMR δ 4.28 (1H, s, H-3), 5.89 (1H, d, J = 4.7 Hz, H-6), 5.04 (1H, m, H-16), 1.10 (3H, s, H-18), 1.22 (3H, s, H-19), 1.53 (3H, s, H-21), 7.38 (1H, d, J = 15.8 Hz, H-23), 7.42 (1H, d, J = 15.8 Hz, H-24), 1.51 (3H, s, H-26), 1.52 (3H, s, H-27), 1.49 (3H, s, H-28), 1.24 (3H, s, H-29), 1.62 (3H, s, H-30), 1.93 (3H, s, $COCH_3$); ^{13}C-NMR δ: 40.3 (C-1), 211.3 (C-2), 82.1 (C-3), 47.3 (C-4), 139.8 (C-5), 122.0 (C-6), 24.6 (C-7), 43.5 (C-8), 49.7 (C-9), 35.9 (C-10), 213.1 (C-11), 49.3 (C-12), 49.1 (C-13), 51.4 (C-14), 46.8 (C-15), 80.6 (C-16), 59.4 (C-17), 20.4 (C-18), 19.3 (C-19), 81.5 (C-20), 26.0 (C-21), 215.5 (C-22), 32.6 (C-23), 37.0 (C-24), 70.8 (C-25), 26.4 (C-26), 26.5 (C-27), 22.7 (C-28), 25.0 (C-29), 20.9 (C-30), 170.6 ($COCH_3$), 22.4 ($COCH_3$).
